# Threshold in North Atlantic-Arctic Ocean circulation controlled by the subsidence of the Greenland-Scotland Ridge

**DOI:** 10.1038/ncomms15681

**Published:** 2017-06-05

**Authors:** Michael Stärz, Wilfried Jokat, Gregor Knorr, Gerrit Lohmann

**Affiliations:** 1 Alfred Wegener Institute Helmholtz Centre for Polar and Marine Research, Am Handelshafen 12, Bremerhaven 27570, Germany; 2 School of Earth and Ocean Sciences, Cardiff University, Cardiff CF10 3AT, UK

## Abstract

High latitude ocean gateway changes are thought to play a key role in Cenozoic climate evolution. However, the underlying ocean dynamics are poorly understood. Here we use a fully coupled atmosphere-ocean model to investigate the effect of ocean gateway formation that is associated with the subsidence of the Greenland–Scotland Ridge. We find a threshold in sill depth (∼50 m) that is linked to the influence of wind mixing. Sill depth changes within the wind mixed layer establish lagoonal and estuarine conditions with limited exchange across the sill resulting in brackish or even fresher Arctic conditions. Close to the threshold the ocean regime is highly sensitive to changes in atmospheric CO_2_ and the associated modulation in the hydrological cycle. For larger sill depths a bi-directional flow regime across the ridge develops, providing a baseline for the final step towards the establishment of a modern prototype North Atlantic-Arctic water exchange.

## Abstract (web summary)

During the Cenozoic the Arctic Ocean change from a restricted freshwater regime towards more saline modern ocean conditions is supposedly driven by the subsidence of the Greenland-Scotland Ridge. Here, the authors derive a threshold for this shift, constrained by the characteristic depth of wind mixing.

The tectonic evolution of ocean gateways and CO_2_ changes are key controls of Cenozoic (from 65 Myr ago until present) climate change and ocean circulation. The last 65 Myr ago (Ma) of the earth's history are characterized by a gradual long-term cooling trend, and the superposition of relatively abrupt climate changes that occurred on much faster timescales^1^,^2^,^3^. However, it remains a major challenge to identify to which extent tectonic events and CO_2_ changes controlled the different trends and climate variations.

Especially during the late Eocene to early Miocene interval (∼35–16 Ma), the climate of the North Atlantic-Arctic sector is prone to instabilities^4^,^5^,^6^,^7^,^8^,^9^. Therein, the subsidence of the Greenland–Scotland Ridge (GSR) from subaerial conditions towards a submarine rise constitutes an active ocean gateway control of North Atlantic-Arctic water exchange^9^,^10^,^11^,^12^,^13^. The long-term subsidence history of the GSR is, however, interfered by recurrent Icelandic mantle plume activity, causing topographic uplift in response to thermal variations in the mantle^13^,^14^. Periodic uplifting of the seafloor through the Neogene, driven by frequent mantle plume intensity, has shown to correlate with excursions in Atlantic deep sea δ^13^C records indicating a moderated southward sill overflow of Northern Component Water—a predecessor of modern North Atlantic Deep Water^13^,^15^.

Although the long-term evolution of such ocean gateway developments on adjacent ocean water mass characteristics are generally accepted to induce basin-scale reorganizations^6^,^9^,^12^,^13^,^16^,^17^, the climatic impacts, as well as the associated mechanisms of climate changes remain largely elusive. Using a fully coupled Earth System Model (ESM)^18^, we investigate the effect of the GSR subsidence during an interval between ∼35 and 16 Ma. In our simulations we use Miocene background climate conditions (∼20–15 Ma), as a basis and apply different GSR depths and CO_2_ concentrations as a surrogate for different conditions during the subsidence interval.

We find a non-linear impact of ocean gateway depth controls on the water mass exchange and Arctic Ocean circulation that is mainly controlled by the effect of sill depth on mixed layer characteristics. For gateway depths close to the depth of the mixed layer, additional simulations of different atmospheric CO_2_ concentrations show a modulation of the atmospheric hydrological cycle, controlling the overall Arctic salinity and ocean gyre circulation in the sub-polar Arctic (Greenland and Norwegian Seas). The critical threshold in gateway depth is constrained by the characteristic depth of wind driven mixing, unravelling the underlying processes that allow a theoretical assessment of the circulation system of semi-enclosed ocean basins throughout Earth's history.

## Results

### Experimental approach

**Figure 1:**
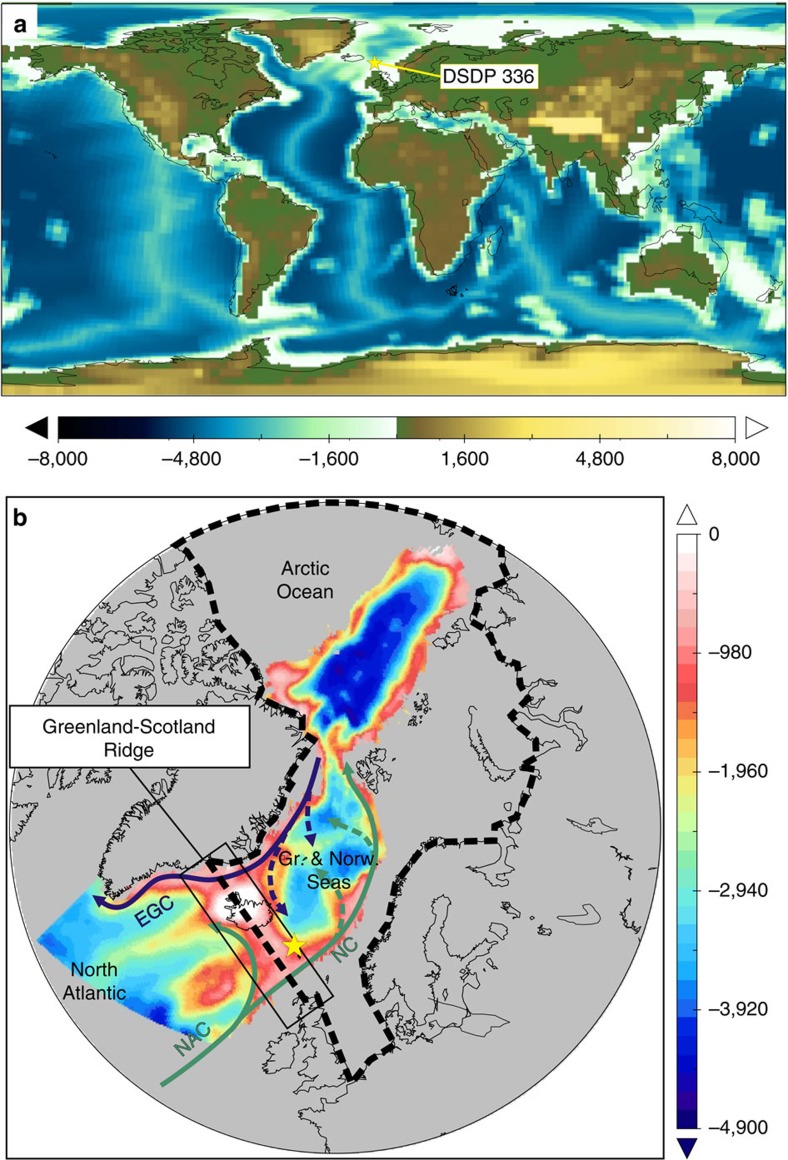
Geographical settings of Miocene topography circa 20 to 15 Ma. (**a**) Global compilation of Miocene geography^19^ (elevation and depth in metres); (**b**) A high resolution (0.5°; depth in metres) bathymetric dataset is embedded in the global setup comprising the northern North Atlantic, subpolar Arctic (Greenland and Norwegian Seas) and the Eurasian Basin in the Arctic Ocean^20^. The Arctic Ocean is defined by the central Arctic and the Greenland and Norwegian Seas as shown by the stippled lines. The schematic circulation shows pathways of the North Atlantic Current (NAC), the Norwegian Current (NC) and the East Greenland Current (EGC). The yellow star reflects the location of site 336 of the Deep Sea Drilling Project^21^ at the northern flank of the GSR.

In this study we apply an ESM (see model description in Methods) to simulate the subsidence of the GSR by incremental changes of the mean ridge height, starting from a quasi-enclosed towards a deep Miocene topographic configuration of the Greenland–Scotland ocean gateway (Fig. 1). The ocean component of the ESM is characterized by a curve-linear grid that provides a maximum horizontal resolution of ∼30 km near the grid pole at Greenland^18^. This ocean grid space is too coarse to resolve non-rotational meso-scale flow patterns, as defined by the internal Rossby radius of deformation. However, in our model the GSR gateway is wide enough to simulate a rotationally controlled flow regime across the gateway. Related to considerable uncertainties of the GSR subsidence history, the model is setup with alternative boundary conditions (early Miocene ∼20–15 Ma)^19^ compared with present-day representing a template used in our ocean gateway studies (for details on the model scenarios and boundary conditions, see Methods). This setting includes a closed Bering Strait and Canadian Archipelago configuration, providing a single ocean gateway control of the GSR. The final model setup is further advanced by embedding a high resolution bathymetry reconstruction of the northern North Atlantic-Arctic Ocean^20^ into the global topographic dataset (Fig. 1).

**Figure 2:**
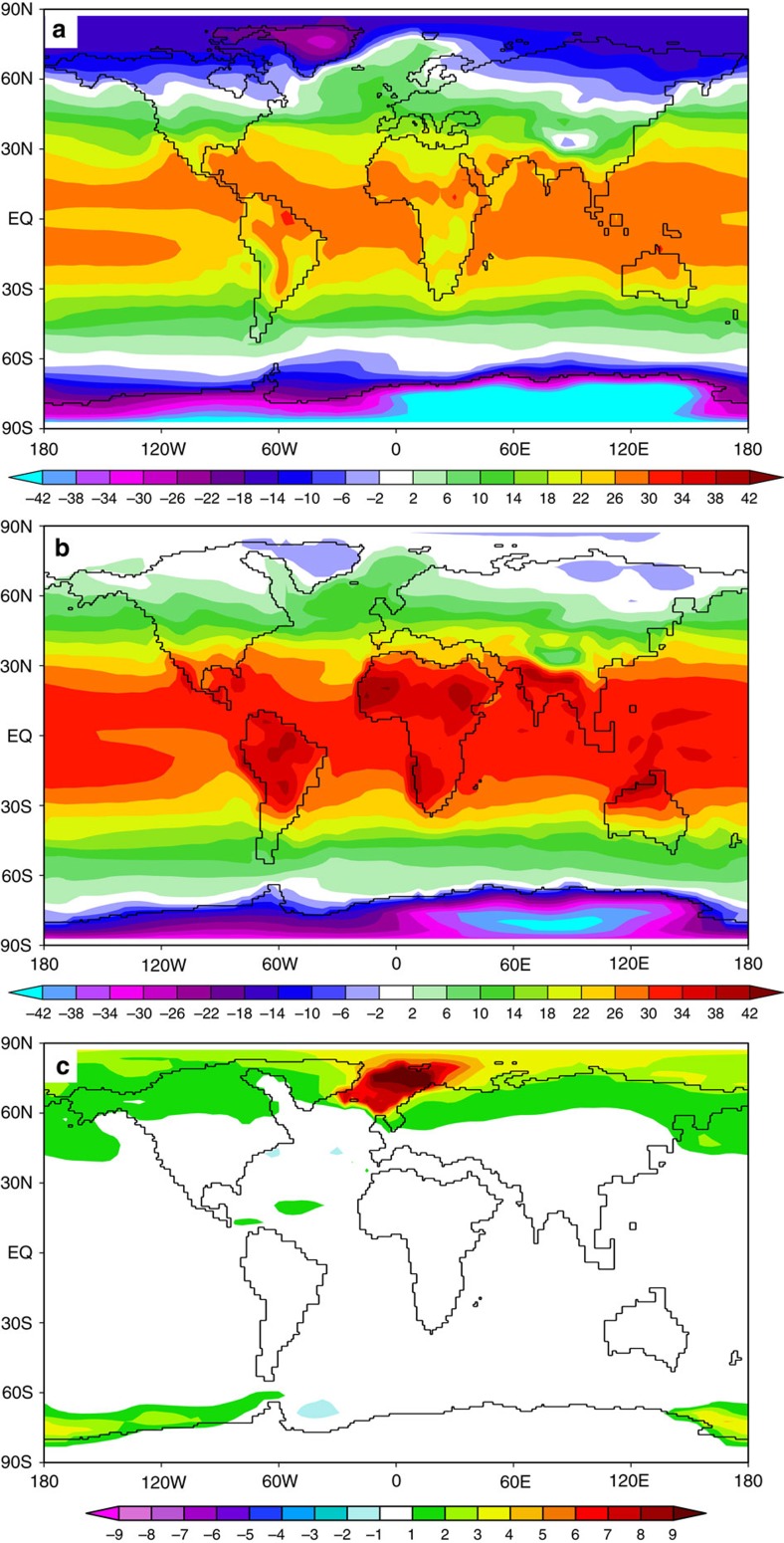
Modelled climatologies of surface air temperatures. Surface air temperatures (SAT in °C) for (**a**) the preindustrial, (**b**) the time interval spanning 35–16 Ma and (**c**) the SAT response to the opening of the Greenland–Scotland Ridge gateway.

**Figure 3:**
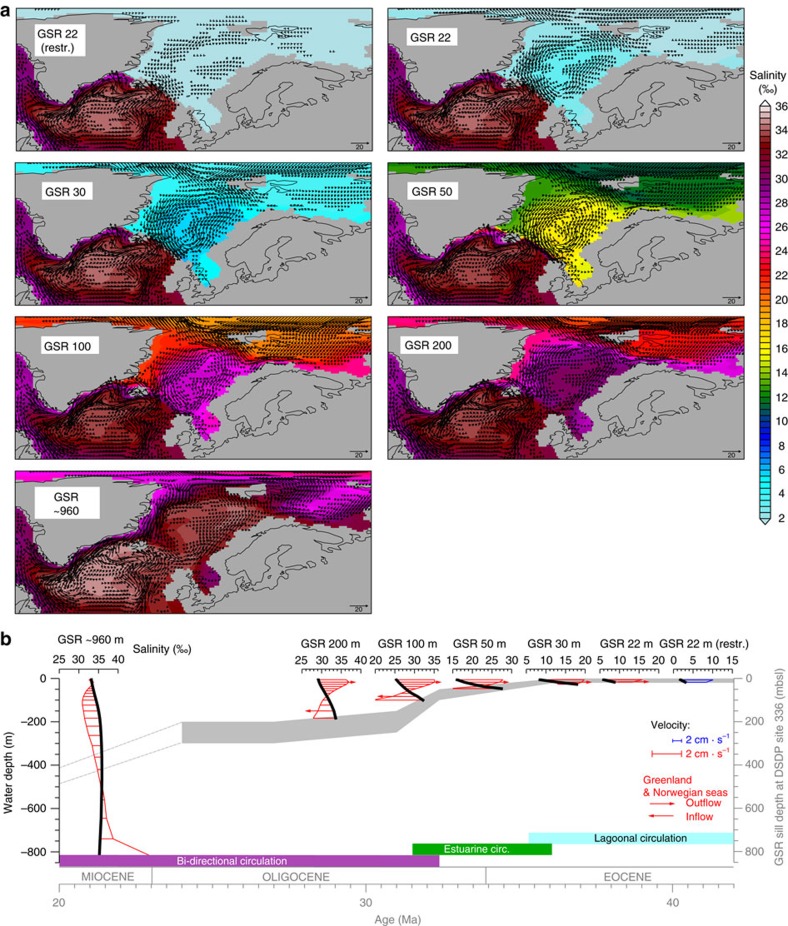
Seaway opening evolution for the last 42 Ma in context of the Greenland–Scotland Ridge subsidence history. (**a**) Salinity (‰) and ocean velocity (cm s^−1^; velocities <0.5 cm s^−1^ are not shown) maps at water depths of 50 m for the model scenarios (EO) at different sill depths of the Greenland–Scotland Ridge (GSR). (**b**) Modelled salinity and velocity profiles across the GSR section (Fig. 1) for the preindustrial (PI) and Miocene GSR sill depths are displayed in context of the subsidence evolution as derived from DSDP site 336 (refs 21, 22).

**Figure 4:**
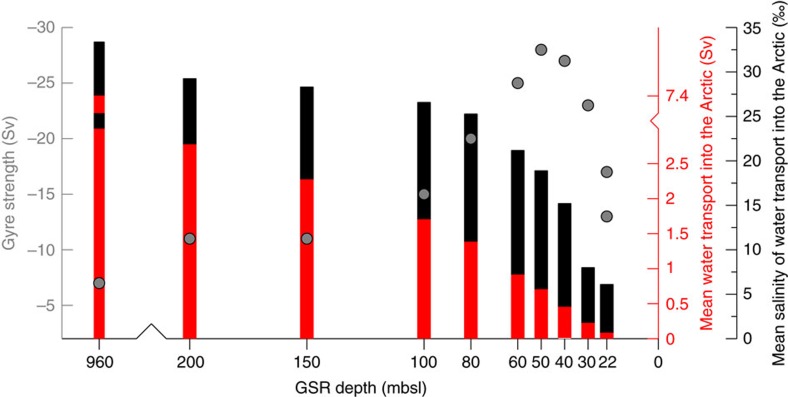
Non-linear response of Greenland-Scotland Ridge sill depth changes on ocean characteristics. The Greenland-Scotland Ridge (GSR) deepening (mbsl, metres below sea-level) scales with a non-linear increase in mean salinity (‰, black bars) and water mass (Sv, red bars) import into the Arctic, whereas a local maximum in gyre strength (grey dots) is obtained by the circulation in the Greenland and Norwegian Seas, as indicated by the horizontal barotropic streamfunction (Sv).

**Figure 5:**
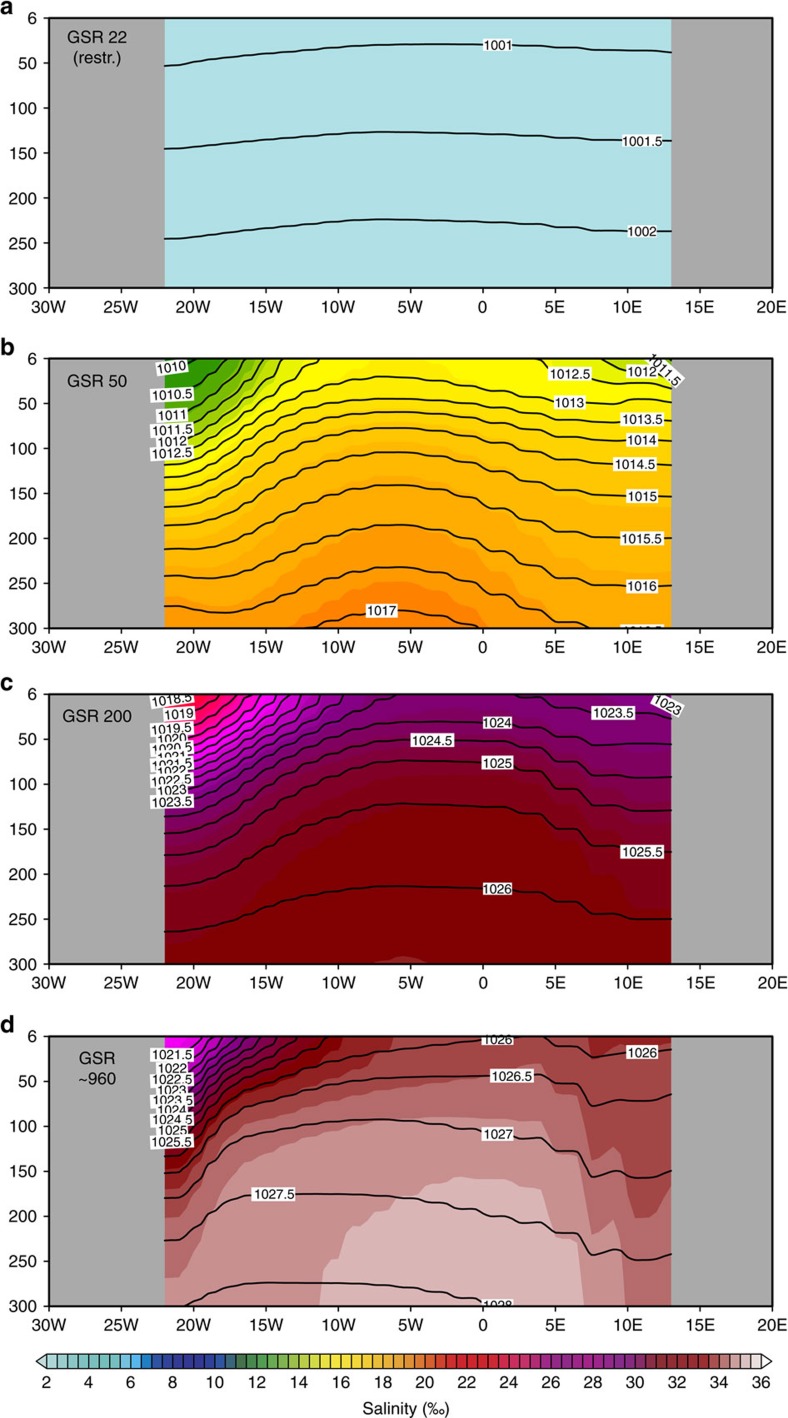
Evolution of the subpolar Arctic gyre from restricted freshwater conditions towards a brackish and modern prototype salinity distribution. Zonal section at ∼70°N across the subpolar Arctic (Greenland and Norwegian Seas) gyre displays the salinity (colour coded; ‰) and pressure (contour lines; Pa) evolution from (**a**) a restricted (sill depth at 22 m) to (**b**) semi-enclosed estuarine (GSR sill depth at 50 m) and (**c**) bi-directional (sill depth at 200 m) to (**d**) modern prototype circulation regime (sill depth at ca. 960 m) across the Greenland-Scotland Ridge gateway.

**Figure 6:**
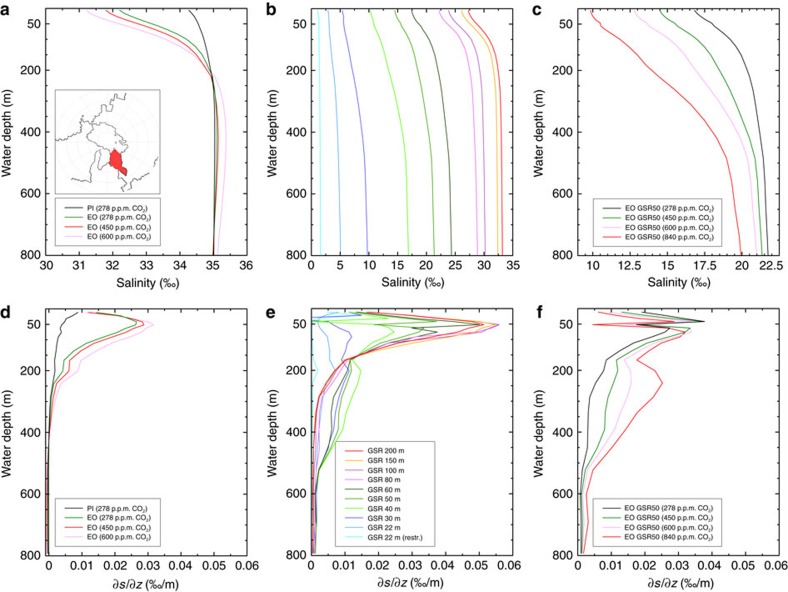
Impact of atmospheric CO_2_ changes and gateway changes on vertical salinity characteristics in the subpolar Arctic. Mean salinity profiles (‰) and haloclines (δ*S*/δ*z*; ‰/m) of the subpolar Arctic (Greenland and Norwegian Seas) for different atmospheric CO_2_ levels (**a**,**d**), Greenland-Scotland Ridge (GSR) gateway sill depths (**b**,**e**) and different atmospheric CO_2_ levels at limited GSR sill depths of 50 metres below sea level (**c**,**f**).

Within a set of model scenarios we consider a gradual deepening of the GSR by stepwise changes (between 22 and 200 m below sea level, mbsl; see Methods and Supplementary Table 1) to study the effect of sill depth changes^21^,^22^ on climate and ocean circulation (Figs 2 and 3). Parallel with the GSR deepening, the corresponding salt water import across the seaway largely controls the overall salinity, baroclinity and gyre strength in the Arctic Ocean (Figs 4, 5 and 6; Supplementary Fig. 5). To analyse the impact of GSR sill depth changes, we primarily focus on the evolution of ocean gateway circulation, the establishment of salinity (density) gradients and the gyre circulation in the Greenland and Norwegian Seas.

### Lagoonal circulation

The restricted ocean gateway geometry (GSR sill depth at 22 mbsl and GSR width of ∼370 km, as compared with our standard gateway width of ∼1,300 km) results in a quasi-enclosed Arctic Ocean with minor communication to the world oceans via lagoonal circulation. This circulation is characterized by hydraulic controls of an intense uni-directed flow regime (Fig. 3b) that is accomplished by a positive virtual balance of the net Arctic freshwater input (net precipitation and river runoff: +0.7 Sv). Thereby, the absence of northward ocean heat and salt transports governs near freezing-point temperatures, near basin wide seasonal sea ice cover and the presence of ephemeral perennial sea-ice (Supplementary Figs 2,4 and 5). Arctic freshwater excess and the reduction of northward ocean heat and salt transport results in an Arctic ‘freshwater lake' stage, accompanied by a regional surface air temperature drop of ∼5–9 °C and decreased precipitation in the Norwegian and Greenland Seas, as compared with the standard model climatology (Fig. 2, Supplementary Fig. 5). In this setting the GSR operates as an oceanographic barrier that steers major parts of North Atlantic Current (NAC) along the isobaths towards Irminger and Labrador Seas (Fig. 3a). The absence of southern placed sources of salty waters that are usually transported by the modern Norwegian Current (NC) analogue inhibits the development of pronounced vertical and horizontal salinity gradients in the Arctic Ocean. Without vertical and horizontal salinity gradients, as provided by a restricted Arctic freshwater environment, the prevailing barotropic mode inhibits a dynamic ocean regime due to minor salinity driven density and pressure gradient forces (Fig. 3).

To highlight the relevance of vertical and horizontal salinity gradients driving Arctic Ocean dynamics, we run an additional model sensitivity study, assuming Arctic water masses of constant salinity (28‰; herein the salinity driven part of the density calculation is kept constant but pressure and temperature related density changes are taken into account). As shown by the sensitivity study, the absence of salinity contrasts minimizes pressure gradients that fail to balance the wind driven Ekman transport, hence the baroclinic geostrophic imbalance results in a collapse of the Arctic Ocean circulation (Supplementary Fig. 6).

A more quantitative approach to analyse the dynamics in the Norwegian and Greenland Seas is given by the calculation of gyre strength—as expressed in terms of the horizontal barotropic streamfunction (vertically integrated water mass movement)—reveals a relative weak gyre strength of −13 Sv within an Arctic freshwater environment. Resulting pressure gradient forces, which are typically associated with ocean baroclinity, are largely impeded—thereby the gyre strength is strongly suppressed. Remaining gyre dynamics within the quasi-enclosed Arctic Ocean basin are mainly forced by wind-stress.

### Semi-enclosed estuarine circulation

In general, progressive GSR gateway deepening from 22 m to ∼80 m enables the northward penetration of dense North Atlantic waters via near bottom flow across a shallow GSR sill establishing a semi-enclosed estuarine circulation. For characteristic gateway depths that are placed within the typical depth range of the Ekman layer, the ingress of North Atlantic water to the Greenland and Norwegian Seas is constrained by the opposed Arctic outflow at the surface mixed layer. Thereby, frictional processes at the bottom of the gateway and internal friction between the two water masses limit the inflow of North Atlantic water to the Arctic Ocean.

The inflow of Atlantic water induces a salinisation process towards a brackish Arctic Ocean regime (Fig. 3a,b, Supplementary Fig. 5). As a result of ocean salt exchange across the GSR gateway (Fig. 4) and net Arctic freshwater input via the atmospheric hydrological cycle (net precipitation and river runoff), a vertical Arctic salinity gradient and halocline establishes for the first time (Fig. 6b,e). Thereby the location of the strongest vertical salinity (density) gradient (Fig. 6) defines the depth of the halocline (a similar depth of the pycnocline is given in Supplementary Fig. 14). The formation of horizontal and vertical salinity gradients invigorate Arctic gyre circulation following isolines of salinity. Highest densities at the surface are generated by Ekman mixing and outcropping isopycnals in the centre of the gyre (Fig. 5). To maintain this gyre circulation, the corresponding horizontal density gradient must be established to obtain the compensation of the wind driven Ekman transport (baroclinic geostrophic balance)^23^. Wind stress and associated Ekman mixing (generally operating in the upper ca. 40–200 m of the ocean)^23^ induces wind stirring and relative buoyancy of dense subsurface waters that in turn establish the corresponding horizontal pressure gradient across the gyre. Lateral entrainment of subsurface waters from the GSR gateway conserves mass transport for this upwelling.

For a detailed perspective on the dynamics of a semi-enclosed estuarine circulation, we further subdivide the circulation regime into two flow cases that are defined by the relative depth of the ocean gateway with respect to the mixed layer depth. For GSR sill depths between 22 and 50 mbsl (first case), limited inflow of Atlantic water to the Arctic occurs above the characteristic depth of the mixed layer (∼50 mbsl) and for the second flow case, the bulk inflow of Atlantic water takes place beneath the surface mixed layer at gateway depths between 50 and 80 mbsl.

At GSR depths between 22 and 50 mbsl, the first case depicts a bulk inflow of Atlantic water that takes place above the characteristic depth of the surface mixed layer (∼50 mbsl). Thereby, the lateral inflow of North Atlantic waters across the gateway perturbs the Arctic stratification as represented by excursions of the characteristic halocline (Fig. 6e). Notably, at 50 mbsl of GSR sill depth, the gyre strength approaches its maximum at −28 Sv, matching the modelled halocline (Fig. 6d,e) and the depth of the surface mixed layer (∼50 mbsl, see depth of the wind driven upper ocean layer provided in Methods). At gateway sill depths that intersect the approximate depth of the mixed layer, the injection of salty Atlantic water just below the halocline establishes pronounced vertical salinity contrasts. This corresponds to effective Ekman pumping and steep baroclinity, obtaining intensified Arctic gyre strength (Fig. 4; −28 Sv). The second case sets GSR sill depths between 50 and 80 mbsl with bulk inflow of Atlantic that places beneath the mixed layer depth. The vertical separation of bulk Atlantic water inflow with respect to the mixed layer above tends to weaken the amplitude of the halocline and therefore the baroclinic-geostrophic balance of the gyre circulation.

### Bi-directional to modern prototype circulation

By deepening the GSR sill depth from 80 to 100 mbsl we identify the transition from an estuarine towards a bi-directional seaway circulation and a ventilation of the Arctic Ocean (Fig. 3). The transition regime is controlled by the depth of the halocline, defining the interface between the surface mixed layer and the ocean layer below.

The subsidence of the GSR sill from the surface mixed layer to the ocean layer underneath, as defined by the characteristic depth of the halocline (∼50 mbsl, see also pycnocline in Supplementary Fig. 14), obtains vertical differentiation of the surface-subsurface outflow and underlying inflow to the Arctic Ocean. At gateway depths that are placed well beneath the depth of the Arctic halocline, unrestricted Atlantic water inflow to the sub-polar Arctic is indicated by a pronounced unperturbed Arctic halocline. In parallel with the establishment of an Arctic halocline (Fig. 6e) and bi-directional circulation regime, the through-flow into the Arctic Ocean constitutes the reorganization from a brackish towards ventilated Arctic salinity regime. A reduction in strength of the Arctic gyre is compensated by the evolution of a north-south directed current system instead (Fig. 4).

Progressive deepening of the GSR sill from 100 to 200 mbsl towards a deep gateway configuration of ∼960 m depth additionally strengthens the entrainment of Atlantic waters. In return, a considerably decreased Arctic gyre circulation evolves that responds to a more effective cross-sectional gateway transport (Fig. 4). In contrast to a bi-directional circulation across the gateway, the final establishment towards a modern prototype current system is characterized by the zonal differentiation between the northward directed North Atlantic Current in the East and the East Greenland Current to the West (NAC versus EGC) of the Greenland and Norwegian Seas (Fig. 3). Although a modern like deep GSR gateway configuration provides unrestricted ocean water interchange and therefore reducing the Arctic halocline, however, our gateway studies still obtain stronger than preindustrial vertical salinity contrasts (Fig. 6a). This is mainly due to relatively fresh Arctic surface waters—fed by Arctic rivers and net precipitation—balanced by relatively salty southern sourced Atlantic water (for information on the modelled freshwater balance and salinity trends in the Arctic Ocean, see Methods).

### Atmospheric CO_2_ controls on the Arctic Ocean regime

**Figure 7:**
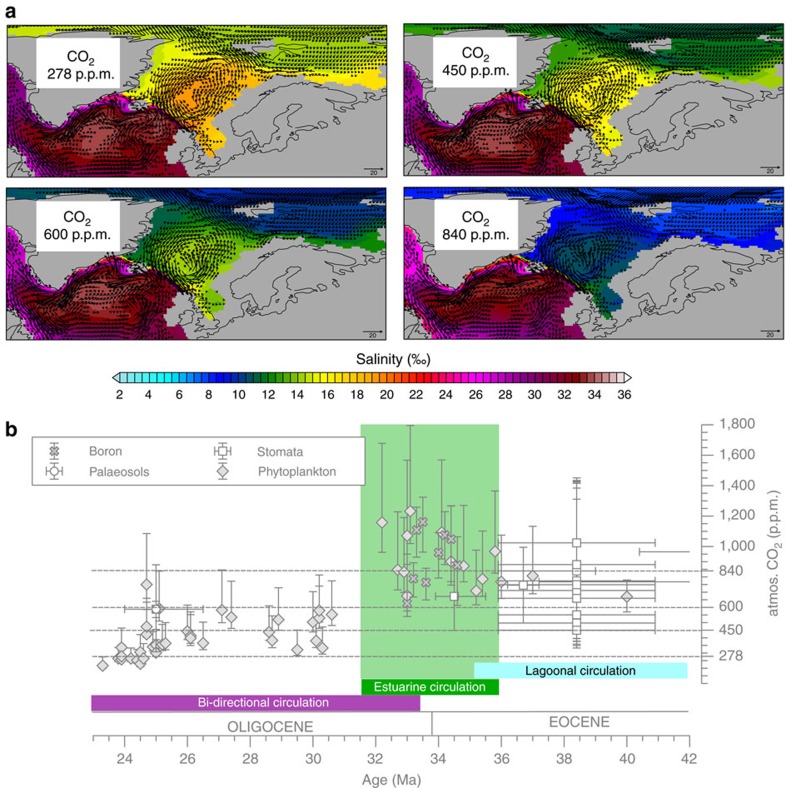
North Atlantic-Arctic circulation regime modulated by atmospheric CO_2_ levels at sensible Greenland-Scotland Ridge sill depths for circa 36 to 31.5 Ma. (**a**) Salinity (‰) and ocean velocity (cm s^−1^; velocities <0.5 cm s^−1^ are not shown) maps at water depths of 50 metres below sea-level (mbsl) for model scenarios (sill depth at ∼50 mbsl) at 278, 450, 600, 840 p.p.m. CO_2_ in the atmosphere, respectively. (**b**) Evolution of Greenland-Scotland Ridge gateway circulation and different atmospheric CO_2_ levels set into context of reconstructed CO_2_ proxy history (error bars show documented uncertainties)^25^,^26^.

To further test the sensitivity of stratification and gyre strength at the brackish salinity regime (GSR sill depth at 50 m), we focus on the effect of different atmospheric CO_2_ concentrations, capturing a wide range of Eocene to Miocene greenhouse gas variations (∼278–1,200 p.p.m. in the atmosphere, refs 24, 25, 26, Fig. 7). Therefore, we additionally investigate the model scenario at critical GSR depths (∼50 mbsl) by a variety of atmospheric CO_2_ concentrations (278, 450, 600, 840 p.p.m.). The CO_2_ concentration case at 278 p.p.m. reflects the climate sensitivity at preindustrial CO_2_ levels, whereas the standard CO_2_ levels at 450 p.p.m. represents the modelled climatology (Fig. 2b) that has also been used for the previously presented gateway studies.

At fixed gateway depths (GSR sill depth at 50 mbsl), providing a semi-enclosed estuarine circulation, we find that CO_2_ controls via the atmospheric hydrological cycle modulate the strength in Arctic gyre circulation (Supplementary Fig. 7). Elevated atmospheric CO_2_ levels induce an increase in the Arctic freshwater budget (Supplementary Table 2) and a more accentuated halocline establishes in the Arctic Ocean (Fig. 6). Increasing freshwater excess in combination with reduced Atlantic water inflow progressively shifts Arctic salinities towards fresher conditions. Interestingly, at fixed gateway depths of ∼50 mbsl the standard CO_2_ case (450 p.p.m.) reveals maximum gyre strength. Especially in this CO_2_ scenario a pronounced Arctic halocline (pycnocline) provides pronounced baroclinic forcing to balance the wind stress (for further information on the effect of atmospheric CO_2_ changes on the brackish Arctic Ocean see Methods).

## Discussions

The modelled climate shows distinct global warming (Fig. 2) that matches the global mean temperature reconstruction suggesting +6 °C increase of surface temperatures^27^ with respect to preindustrial conditions. Apart from topographic height reduction associated with the lapse rate, major warming anomalies as compared to preindustrial (Supplementary Fig. 1) are related to changes in atmospheric CO_2_ and changes in land surface characteristics (land albedo, potential evaporation over land) related to aspects of the global energy balance^28^,^29^ (planetary albedo, effective longwave emissivity). Our modelled Paleogene climate is characterized by warm background conditions and exhibits a sensitive surface air temperature response to CO_2_ perturbations, which is governed by climate feedbacks, such as water vapour^30^ (+58% increase compared to preindustrial; Supplementary Table 2) and changes from single to multi-year sea-ice^28^,^29^,^30^ (Supplementary Fig. 2). Previous investigations show that the model generally reveals a strong climate response due to high sensitivity of climate feedbacks especially in the lower range of CO_2_ changes (between 278 and 450 p.p.m. CO_2_)^28^,^31^. Compared with preindustrial, the computed Paleogene climate shows a reduction in sea-ice volume, increased water vapour, precipitation and river runoff (Supplementary Fig. 3 and Supplementary Table 2), consistent with precipitation records^32^, proposing a stronger Arctic Ocean freshwater balance (further information is given in modelled freshwater balance and salinity trends in the Arctic Ocean in Methods).

Geological constraints on the subsidence history of the GSR from a subaerial gateway towards modern sill depths remain largely elusive. Early Deep Sea Drilling Project (DSDP) reconstructions of paleo-water depth^21^,^22^ at the GSR do not suggest significant tectonic activity until 36 Ma ago. Thereafter, accelerated gateway deepening across the Eocene-Oligocene transition to depth ranges ∼200–300 mbsl is followed by a prolonged period of tectonic dormancy. The superposition of Icelandic mantle plume variability and associated seafloor uplift variations through time provides an in detail unknown control on the GSR gateway opening. Seismic reflection profiles that transect the V-shaped Reykjanes Ridge south of Iceland offer insight into the temporal evolution of Icelandic mantle plume activity up to 55 Ma back in time^14^: reconstructed mantle plume activity before initial GSR subsidence (>36 Ma), as derived from residual depth anomalies of seismic profiles indicate a strong decline in Icelandic mantle plume activity between ∼55 and 35 Ma, but with a still subaerial ridge at the end of this period. Significant deepening and the subsidence of the GSR below sea level afterwards sets an active control of modest periodic (3–8 Ma period) mantle plume variations that ranges within the uncertainties of depth reconstructions^9^,^10^,^11^,^12^, response in depth variations^14^,^15^ and sea level fluctuations^33^.

Further to the North of the GSR, tectonic widening of the Fram Strait constitutes an alternative candidate that complicates the overall interpretation on the gateway opening of the central Arctic Ocean^5^,^20^. Although the transition from anoxic towards fully oxygenated conditions found in sediments records in proximity to the North Pole suggests a ventilation control via the Fram Strait, however, age model interpretations remain ambiguous^5^,^34^,^35^.

As suggested by the geological subsidence model of DSDP site 336 (ref. 21), before initial gateway deepening around 36 Ma, lagoonal circulation conveyed Arctic freshwater excess towards the North Atlantic. For this period a relatively warm climate likely enhances Arctic freshwater excess that promotes a sluggish freshwater environment in the restricted Arctic Ocean. As a result of the applied boundary conditions, our model shows a barotropic Arctic Ocean that fails to establish sufficient pressure gradient forces to balance the wind driven geostrophic circulation. Such a resilient Arctic Ocean circulation does not support the development of contourite drift deposits^36^ before the gateway opening, enabling a regime of ultra-slow sedimentation rates such as suggested for quasi-enclosed ocean basins^34^,^35^.

Referring to the GSR depth record, accelerated subsidence of the GSR around 36–31 Ma initiates a semi-enclosed estuarine seaway exchange and brackish salinity regime in the sub-polar Arctic. In response to the GSR deepening, a nonlinear salinisation process controls the stratification and the associated baroclinic geostrophic balance in ocean circulation, which in turn coincides with initial contourite sediment drift formation in the Greenland and Norwegian Seas^12^,^37^. Within time periods of characteristic gateway depth levels that are placed around 50 mbsl, our model results indicate an accelerated circulation regime with most pronounced baroclinic forces driving the gyre circulation. On the basis of the GSR depth record, our results suggest a change from semi-enclosed estuarine towards a bidirectional circulation ∼32 Ma, induced by subsidence of the GSR beneath the surface ocean mixed layer. Such a scenario matches the initial change in isotope records at the Walvis Ridge^9^,^38^—a proxy used for identifying the origin of water masses. This record indicates the contemporary onset of sub-polar Arctic deep water reaching the South Atlantic around 33 Ma (Supplementary Information and Supplementary Fig. 8). Accompanied by the salinisation process, mixing of a δ^18^O depleted ‘Arctic freshwater lake' with the surrounding oceans implies changes in the global salinity distribution and global shifts in benthic δ^18^O (Supplementary Table 2), which lies within the variability of compiled isotope records^1^,^2^,^3^.

In combination, the GSR history and the model results suggest a period (∼36–32 Ma; Fig. 3b) of estuarine North Atlantic-Arctic circulation across the Eocene-Oligocene transition (∼33.8 Ma) that is characterized by remarkable CO_2_ variations^26^,^39^ and relative sea-level changes^33^ (Fig. 6b and Supplementary Fig. 8). Our results suggest that after a first order tectonic pre-conditioning of the GSR gateway and the establishment of an estuarine circulation, atmospheric CO_2_ changes and glaciation induced sea level variations may have modulated the overall salinity and gyre strength in a brackish Arctic Ocean at shorter millennial time-scales. Especially at the Eocene-Oligocene transition, the contemporary drop in atmospheric CO_2_ and relative sea level changes with respect to the GSR sill depth (for example, by Antarctic glaciation) may have partly counterbalanced the opposing effects of the Arctic freshwater balance and the Atlantic water inflow on the salinity and circulation of the Arctic Ocean. According to the GSR subsidence record^21^,^22^, at ∼32 Ma the GSR sill falls well below the surface–subsurface ocean interface (>∼50 mbsl) as defined by the halocline, which constitutes the establishment of a bidirectional seaway circulation and the development towards a present-day north-south directed EGC-NAC current system.

In principal, our modelling study shows a pronounced near surface stratification that defines the critical depth in ocean gateway circulation of a semi-enclosed Arctic Ocean basin. The dynamic exchange across the gateway is fundamentally limited to the characteristic depth of wind driven mixing. This depth is determined by the depth of frictional influence, which is controlled by the Coriolis force and local ocean conditions like stratification and turbulent mixing. In light of our modelling results, the theoretical derivation on the depth of frictional influence restricts the critical threshold regime in ocean gateway circulation to the wind driven upper ocean layer. This theoretical and dynamical framework provides a baseline to derive critical gateway depths that are defined by the transition between a semi-enclosed estuarine and fully ventilated ocean regime.

In future studies, the presented framework gives support to interpret high-resolution sediment records that target past climate variability in the North Atlantic-Arctic sector. Given the lack of calcareous carbonates in sediment records from the Greenland and Norwegian Seas and the Arctic Ocean, an analysis of near bottom flow changes associated with the simulated ocean circulation regime shift via, for example, sortable silt records or oceanic circulation reconstructions using high resolution imaging of sedimentary structures might be sensible. Such methods could be complemented by biomarker based reconstructions of temperature (for example, alkenone SST) and sea ice conditions (for example, IP25) to test the presented framework.

## Methods

### Model description

The General Circulation Model COSMOS (community of earth system models) comprises the standardized IPCC4 model configuration which incorporates the ocean model MPIOM^18^, the ECHAM5 atmosphere model at T31 spherical resolution (∼3.75 × 3.75°) with 19 vertical levels^40^ and the land surface model JSBACH including vegetation dynamics^41^,^42^. The ocean model is resolved at 40 unevenly spaced vertical layers and takes advantage of a curve-linear grid at an average resolution of 3 × 1.8° on the horizontal dimension, which increases towards the grid poles at Greenland and Antarctica (∼30 km). High-resolution in the realm of the grid poles advances the representation of detailed physical processes at locations of deep water formation, as Weddell, Labrador and Greenland and Norwegian Seas. The ocean model includes an Hibler-type dynamic-thermodynamic sea-ice model. The interactive transfer of energy and fluxes between atmosphere and ocean runs without flux corrections and is handled via the coupler OASIS3 (ref. 18). Net precipitated water over land, which is not stored as snow, intercepted water or soil water, is either interpreted as surface runoff or groundwater and is redirected towards the ocean via a high-resolution river routing scheme^43^. The model has been applied for scientific questions focusing on the Quaternary^29^,^44^,^45^,^46^, as well as the Neogene^28^,^31^,^47^,^48^,^49^.

### Model boundary conditions

The model setup uses state-of-the-art model boundary conditions encompassing a time period (∼23–15 Ma) within the early and middle Miocene, which we apply as a template to investigate our North Atlantic/Arctic gateway studies. For this time period the continental ice on Antarctica as well as tectonic boundary conditions (continental distribution, land surface elevation, shelf seas, bathymetry and sediment loading) are derived from Herold *et al*.^19^. In general the Miocene orography (Andes, Rocky Mountains, East Africa, Tibetan Plateau) and the height of the Antarctic ice-sheet are reduced compared to present-day, whereas the Greenland ice-sheet is absent in the Miocene setup. Ocean gateways like Bering Strait and the Canadian Archipelago evolved after the middle Miocene but Tethys through-flow and Panama Seaway still connected the ocean basins. After the closure of Turgay Strait during the middle/late Eocene^50^, the general late Eocene to Miocene ocean gateway settings at the Arctic have been established. Into this global tectonic reconstruction we have nested a regional high resolution bathymetric dataset comprising the middle Miocene (15 Ma) Greenland and Norwegians Seas and Eurasian Basin^20^, which is adequately represented in our spatial model resolution due to the close locality of the grid pole (Fig. 1). The Greenland-Scotland Ridge acts as an oceanographic barrier and represents the single gateway restricting the exchange of water masses/fluxes between the Arctic Ocean (incl. the Greenland and Norwegian Seas) and the northern North Atlantic.

Because of the tectonic opening of the Atlantic basin in time, the depth (∼970 m) and width (∼1,300 km) dimensions of the GSR provided by the Miocene bathymetric model setup are comparably smaller than preindustrial. Further north, the Miocene bathymetric constraints also show a more shallow (∼1,900 m depth) and a more narrow (∼500 km) Fram Strait with respect to the preindustrial bathymetry. The study of Jakobsson *et al*.^5^ suggests that the Fram Strait progressively opened at ∼18.2–17.5 Ma, which is accompanied by a regional Arctic sea-level drop^51^. In contrast, a more recent age model—established by Rhenium–Osmium isotope measurements—indicates that such an opening might have occurred much earlier during the Late Eocene^35^,^36^. Besides conflicting age models^4^,^34^,^35^,^52^, depth reconstructions show a narrow and relatively deep (∼2,000 mbsl) Fram Strait that already existed during the Oligocene (∼30 Ma)^20^. Further, sill depth variations of the Fram Strait during the Oligocene-early Miocene (∼30–20 Ma) possibly ranged between ∼2,000 and 1,500 mbsl, before the Fram Strait progressively broadened between 20 and 15 Ma (ref. 20). Although the sill depth changes (between ∼2,000 and 1,500 mbsl) of the Fram Strait might be important for the exchange of deepwater masses^20^, nevertheless, our study focuses on the effect of GSR sill depth changes in the range of the upper 200 mbsl.

On the basis of a Late Miocene vegetation reconstruction^53^, we derived physical soil characteristics, such as soil albedo and maximum water holding field capacity by adapting vegetation related parameters from Stärz *et al*.^29^. In general the global soil albedo in the Miocene setup decreases in the visible (−0.01) and near infrared spectrum (−0.03) compared to preindustrial (PI; 0.13 and 0.21, respectively). Further the total water holding field capacity increases (+0.03 m) with respect to PI (0.63 m).

The time interval between 35 and 16 Ma is characterized by changes in greenhouse gas concentrations. We performed several experiments with different CO_2_ forcing scenarios (278, 450, 600 and 840 p.p.m.) that are within the range of CO_2_ reconstructions^24^,^25^,^26^,^39^ (Supplementary Table 2).

### Model scenarios

The model scenarios, which have been performed in this study, are listed in the Supplementary Table 1. The preindustrial control run is described in Wei and Lohmann^44^. The final 100 years of each model simulation are used for analysis.

We set-up two model scenarios with Miocene boundary conditions as a paleo template for our experiments starting from present-day ocean salinity and temperature fields with atmospheric CO_2_-levels at 278 (EO_278) and 450 (EO_450) p.p.m. Both scenarios (EO_278, EO_450) run for at least 4,000 yrs in order to minimize salinity and temperature trends in the deep ocean. The effect of tectonic gateway changes (subsidence of the Greenland-Scotland Ridge) on the ocean dynamics is investigated by means of various model scenarios. The ocean gateway sensitivity studies are performed at different height and width dimensions of the GSR. By initializing the ocean model from the final 100 yrs mean of EO_450, we performed model scenarios by changing the GSR sill depth to 200, 150, 100, 80, 60, 50, 40, 30 and 22 mbsl, respectively. In a further model scenario we decrease the width of the GSR ocean passage towards ∼370 km at a sill depth of 22 mbsl (comprising the uppermost two vertical layers of the ocean's model grid) respectively, in order to simulate the effect of an oceanographically quasi-enclosed system of the Arctic Ocean. This strategy allows us to pursue the long-term climate response as a consequence of the tectonic GSR subsidence history at timescales of millions of years and to identify potential nonlinear behaviour in this process. Further we initialized all GSR model scenarios with 1‰ salinity and 0 °C ocean temperature of the Arctic Ocean in order to reach an equilibrated state after another 2,000 yrs of model integration.

Apart of the gateway sensitivity studies we performed additional experiments at different levels of atmospheric CO_2_ (Supplementary Table 1) reflecting a broad range of greenhouse gases representative for the Eocene to Miocene time period (Supplementary Fig. 8).

### Timing constraints

The geological understanding on the subsidence of the Greenland-Scotland Ridge (GSR) during the Eocene towards an oceanographic rise at present water depths remains a major challenge^54^. The general understanding is that the subsidence of the submarine ridge (Fig. 1) controlled the onset (∼35–33 Ma)^9^,^10^,^11^ and long-term variability^13^,^15^ of the Atlantic circulation by southward overflow of Northern Component Water (NCW, which constitutes the precursor of modern North Atlantic Deep Water). In general, we classify two main groups of marine reconstructions that constrain different timing on the opening of the Arctic Ocean:

#### Early Oligocene opening

Palaeontological depth estimates at DSDP site 336 (Figs 1 and 3)^21^,^22^ suggest that during the early Oligocene at 32.4–27 Ma, the rapid deepening of the GSR has been triggered by an abrupt suppression of the Icelandic mantle plume^22^,^55^. This coincides with the onset of deepwater formation as indicated by sediment drift body formation in the northern North Atlantic^10^,^12^ and parallels the onset (∼33 Ma) of decreasing radiogenic neodymium (Nd) isotope composites (^143^Nd/^144^Nd normalized, expressed as ɛ_Nd_ in Supplementary Fig. 8) in the South Atlantic, respectively, which has been interpreted as a signal of NCW derived from the Greenland and Norwegian Seas^9^.

#### Late Oligocene/early Miocene opening

Findings of a major unconformity along the western North Atlantic continental rise have been associated to tectonic controls of deep water outflow from the Greenland and Norwegians Seas during the late Oligocene to earliest Miocene^56^. Moreover, the lack of cosmopolitan benthic foraminifer species^57^,^58^ and indications of poorly ventilated intermediate/deep water^11^, as seen in Oligocene strata from the Norwegian-Greenland Seas, rather point towards the retention of an open circulation regime and restricted oceanographic conditions of the Greenland and Norwegian Seas late into the early Miocene^21^,^22^,^59^.

### Modelled freshwater balance and salinity trends

Although the model reproduces the reconstructed global Miocene temperature signal^27^ (Fig. 2, Supplementary Table 2) some classic model discrepancies still remain, such as relatively warm tropical and cool polar temperatures compared to the proxy records^27^^,^^60^ (Supplementary Fig. 9).

In consequence the model scenarios might also represent a limited increase of atmospheric water transport towards the Arctic region. Although our modelled increase of freshwater transports towards the Arctic still may underestimate the import via the atmospheric hydrological cycle to a certain extent^32^, however, the freshwater input that dominates the stratification at the ocean gateway is sufficiently well captured by our model scenarios (Supplementary Table 2).

Fresh/brackish water conditions in the Arctic Ocean are caused by relatively shallow GSR sill depths (<80 mbsl) that limit the inflow of the North Atlantic Current (Supplementary Figs 4 and 5). Low-salinities in this ‘freshwater lake' environment in the Arctic Ocean are maintained by a net freshwater excess (0.67 Sv) that is strongly increased (268%) compared to the preindustrial Arctic freshwater balance (Supplementary Table 2). The model-data uncertainties regarding the global latitudinal warming (Supplementary Fig. 9) and increases in the modelled atmospheric hydrological cycle are likely to be a robust ‘estimate' during the Cenozoic^61^. The model scenarios show an increased precipitation pattern in the high northern latitudes (Supplementary Fig. 3), which is in general agreement with elevated Neogene precipitation records of the northern hemisphere^32^.

In our model scenarios the opening of a quasi-enclosed Arctic Ocean by the GSR subsidence initiates a salinisation process in the Arctic Ocean. Progressive deepening of the GSR sill depth (ca. 22–100 mbsl) shows that the ridge subsidence causes a gradual nonlinear warming and a salinity increase from fresh/brackish to modern conditions in the Arctic Ocean (Supplementary Fig. 5). A similar scenario would be the temporary presence of fresh Arctic surface waters, known as the ‘Azolla event', that have been controlled by limited oceanic heat/salt exchange with adjacent oceans during the Eocene^62^.

### Wind stress

Although we pointed out the main driving mechanism, other forces that are related to CO_2_ changes, like wind stress and large scale ocean circulation changes (Atlantic Meridional Overturning Circulation) may also affect Arctic circulation (Supplementary Fig. 11, Supplementary Table 2). In general, the associated wind stress is essential to induce Ekman transport for the gyre circulation in the Greenland and Norwegian Seas. Compared to preindustrial, EO_450 shows strong reductions in the Greenland and Norwegian Seas wind system (Supplementary Fig. 11), however, the modern-like meridional NAC versus EGC current system is still maintained.

Referring to the gateway studies, the wind field anomalies with respect to EO_450 are mainly related to increased sea ice cover, as a consequence of limited northward heat transports. However, as seen in Supplementary Fig. 11 the wind stress anomalies do not show remarkable changes among model studies using different gateway depths (model studies with GSR sill depth <100 mbsl). Therefore, based on our model studies, we conclude that both the wind stress and density driven pressure gradients are a prerequisite to establish a gyre circulation, however, variations in the gyre strength are rather controlled by the baroclinic effects, as a consequence of gateway depth changes.

### Salinity driven pressure gradient forces on gyre strength

In another model sensitivity study (based on EO_GSR100) we test the impact of prescribing uniform salinities at 28‰ in an Arctic Ocean environment at GSR sill depths of 100 mbsl. Therefore, the modified model version of the ocean density calculation depends on ocean temperature and pressure applying salinity as a constant (28‰)^63^. Without salinity gradients in the Arctic Ocean, the temperature related part in the density calculation is not sufficient enough to establish horizontal density and pressure gradients in order to drive the Arctic Ocean circulation (Supplementary Fig. 6). As a consequence the geostrophic-baroclinic balance is not maintained and the Arctic Ocean circulation system collapses. In contrast to the model sensitivity study (applying uniform Arctic Ocean salinities at 28‰), the model scenario EO_GSR100 shows ocean currents that strongly follow along with the pycnoclines, suggesting a close relationship between ocean currents and pressure gradient forces (Supplementary Fig. 6).

### Effect of atmospheric CO_2_ changes on the Arctic Ocean

Based on the model scenario EO_GSR50 with GSR sill depth at 50 m, we focus on a range of atmospheric CO_2_ changes (278, 450, 600, 840 p.p.m.), ranging from preindustrial CO_2_ (278 p.p.m.) to 840 p.p.m. in the atmosphere (refs 24, 25, 26, 39; Supplementary Fig. 7). Compared to preindustrial, the 278 CO_2_ case (EO_GSR50_278) reveals +3.1 °C of global warming at the surface as a consequence of changes in the boundary conditions^19^,^29^ and an increased Arctic freshwater balance (Supplementary Table 2). Raising atmospheric CO_2_ levels of the EO model scenarios result in additional warming in parallel with increased freshening in the Arctic Ocean (Supplementary Table 2). This is largely related to the effect of an enhanced Arctic freshwater balance in combination with limited gateway water mass exchanges (Fig. 7 and Supplementary Fig. 5).

The CO_2_-induced climate warming (+4.94 °C more than in the 278 CO_2_ case) drives a reinforced Arctic freshwater balance (+0.16 Sv) via the atmospheric hydrological cycle, which largely shifts the salinity regime further towards fresh conditions in the Arctic. The additional Arctic freshwater release that is transferred into the North Atlantic, combined with increased high latitude warming dampens deepwater formation and results in a consequent slowdown of the Atlantic Meridional Overturning Circulation (ca. 5 Sv compared to PI). At the GSR gateway, especially the additional Arctic freshwater export in combination with attenuated salt import of southern sourced North Atlantic waters reduces the overall salinity and baroclinity in the subpolar Arctic. As a result, at high CO_2_ levels (840 p.p.m.) the Arctic gyre strength strongly reduces (ca. 7 Sv) compared to the standard CO_2_ (450 p.p.m.) case.

### Parameter change effects on the model scenarios

In order to decipher the effect of CO_2_ changes (ΔCO_2_) and GSR sill depth changes (ΔGSR) with respect to the synergy term, we applied a factor separation analysis^64^. The identifiers (Exp. Id) that are used in the following formula refer to the list of model scenarios in Supplementary Table 1:


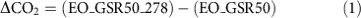



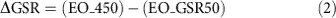



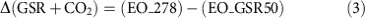



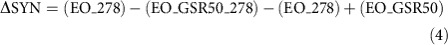


An isolated decline in atmospheric CO_2_ (ΔCO_2_) results in a global atmospheric cooling and a sea surface temperature drop that is most pronounced in regions that are associated with a increase in sea-ice cover (Supplementary Fig. 12). On the other hand, deepening of the GSR sill (ΔGSR) effectively promotes northward directed oceanic heat transport, a change from perennial to seasonal sea ice cover and warming SATs in the high-northern latitudes. The sea surface temperature changes are dominated by atmospheric cooling compared with changes in the heat transport that are associated with oceanic readjustments. The deepening of the GSR sill depth (ΔGSR) from 50 m to Miocene depth reconstructions of ∼960 m provides the establishment of a North/South directed North Atlantic/East Greenland Current system. These changes cause a strong surface air temperature warming in the high northern latitudes that is most pronounced in the Greenland and Norwegian Seas (Supplementary Fig. 12). The comparison of these mono-causal impacts of GSR changes and CO_2_ changes (Δ(GSR+CO_2_)) with their combined effects via the synergy term (ΔSYN) reveals that especially the SAT anomalies in the Norwegian and Greenland Seas are dominated by the associated readjustments in the ocean circulation regime.

Interestingly, in the Southern Ocean both, the ΔCO_2_ drop, as well as ΔGSR deepening, induces a regional SAT warming. However, the combined effect (Δ(GSR+CO_2_)) reveals a general cooling in the Southern Ocean indicating strong feedbacks that are related to the synergy term (ΔSYN).

### Depth of the wind driven upper ocean layer

The thickness of the wind driven layer can be determined by the depth of frictional influence, or approximated by calculation of the Ekman depth:


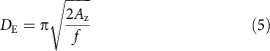


Thereby, *f* constitutes the Coriolis parameter, and the eddy viscosity—a parameter that describes the effect of stratification and turbulent mixing—is given by *A*_z_ (1.0 × 10^−2^ m^2^ s^−1^). *D*_E_ is especially dependent on the choice of the eddy viscosity coefficient *A*_z_. In the numerical ocean model within COSMOS, this parameter is computed for every time step including the speed of ocean currents, background viscosity based on the mixing of internal wave breaking, wind induced stirring dependent on the local static stability (stratification) and the local Richardson number^65^,^66^. Future studies focussing on a more realistic representation of turbulent mixing as given in high resolution meso-scale eddy resolving ocean models, will provide a more detailed assessment of the upper mixed layer of the ocean in order to constrain the regime shift of the GSR gateway studies.

As shown in the manuscript, the depth of frictional influence determines the halocline (pycnocline) and therefore the regime shift in gateway circulation of semi-enclosed stratified ocean basins. Global calculations of the vertical extent of the wind driven layer (except of the low latitudes close to the equator since *f* becomes increasingly low) provide depth values as a function of latitude, between less than 40 m near the poles and more than 200 m at the low latitudes, respectively. The GSR places at ∼58–69°N, yielding calculated Ekman layer depths of ∼46–54 m (Supplementary Fig. 10) in agreement with mixed layer depths in the central Arctic Ocean that range between 25 and 50 m (ref. 67). This result matches the modelled halocline depth—similar results are obtained for the thermocline and pycnocline (Supplementary Figs 13 and 14)—as well as the associated baroclinic geostrophic response with respect to GSR sill depth changes as demonstrated in maximum Arctic gyre strength.

### Extensions of the model code

During sea-ice formation at the surface of the ocean, small amounts of salt water get incorporated into sea-ice. To consider salt water inclusions in the sea-ice matrix, the standard salinity of sea-ice is prescribed at 5‰ in the COSMOS model. For computed sea surface salinities below 5‰, we define new sea-ice salinity at 1‰ in order to account for the formation of sea-ice in a freshwater environment. Further, we included the dynamic computation of the freezing point temperature^68^ which is currently fixed at −1.9 °C. This formula actually integrates the effect of sea surface salinity (*S*; pressure, *ρ*=1,013 hPa) in the calculation of the freezing point temperature *t*_f_:


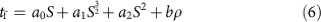



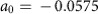



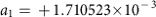



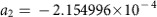



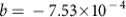


Following the oceanographic opening of the Arctic Ocean gateway and the salinity evolution in the Arctic, as a consequence of salinisation processes the sea surface temperature decreases, which is dominated by the calculation of the freezing point temperature (Supplementary Fig. 4).

### Code availability

The standard model code of the ‘Community Earth System Models' (COSMOS) version COSMOS-landveg r2413 (2009) is available upon request from the ‘Max Planck Institute for Meteorology' in Hamburg (https://www.mpimet.mpg.de).

### Data availability

The modelling data that support the findings of this study are available in Pangaea with the identifier ‘ https://doi.org/10.1594/PANGAEA.873887' (ref. 69).

## Additional information

**How to cite this article:** Stärz, M. *et al*. Threshold in North Atlantic-Arctic Ocean circulation controlled by the subsidence of the Greenland-Scotland Ridge. *Nat. Commun.* **8,** 15681 doi: 10.1038/ncomms15681 (2017).

**Publisher's note:** Springer Nature remains neutral with regard to jurisdictional claims in published maps and institutional affiliations.

## Supplementary Material

Supplementary Information Supplementary Figures, Supplementary Tables, and Supplementary References
