# Supplementary material for: Threshold in North Atlantic-Arctic Ocean circulation controlled by the subsidence of the Greenland-Scotland Ridge
Source: Nat Commun. 2017 Jun 5;8:15681. doi: 10.1038/ncomms15681 (PMC5465373; doi:10.1038/ncomms15681)
Supplement: Supplementary Information — Supplementary Figures, Supplementary Tables, and Supplementary References [file ncomms15681-s1.pdf]

Supplementary Information

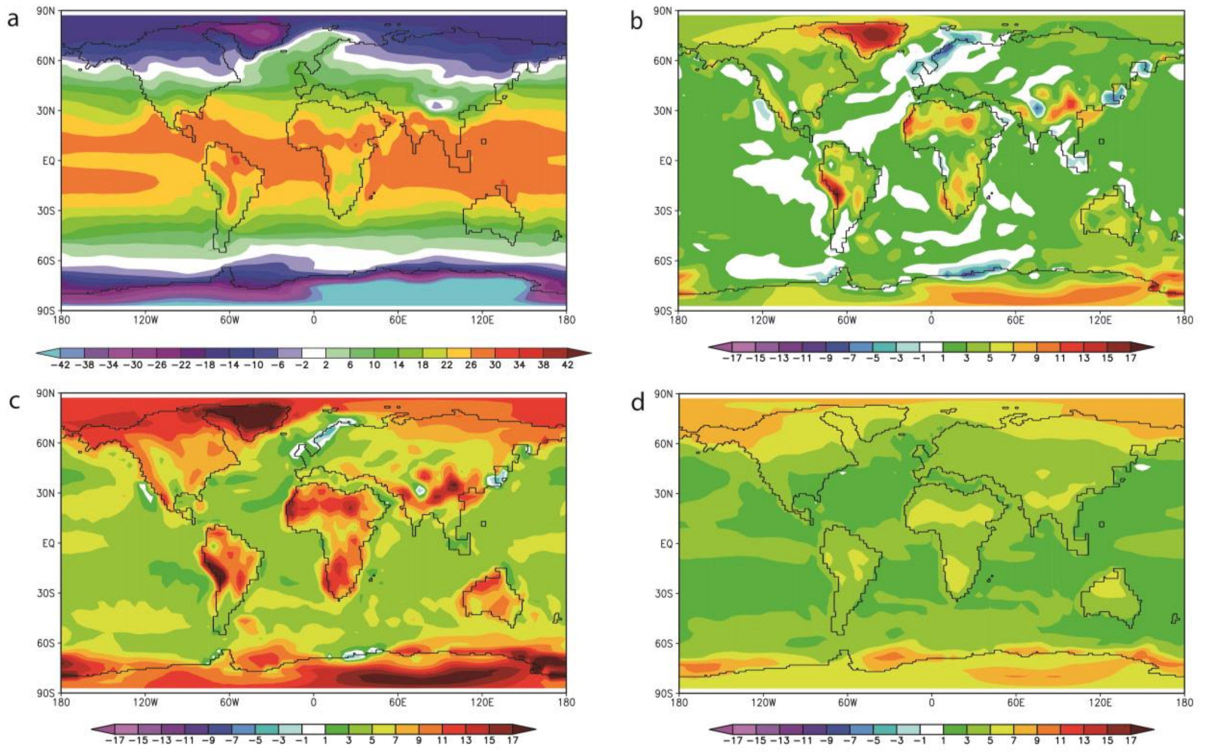

Supplementary Fig. 1: Surface air temperatures of different climate scenarios. (a) Absolute surface air temperatures (SAT in °C) of the preindustrial scenario (PI); (b) EO scenario at low CO_2 (EO_278) compared to PI (EO_278-PI); (c) Miocene scenario at high CO_2 (EO_450) compared to PI (EO_450-PI); (d) Miocene scenario at high CO_2 (EO_450) compared to the Miocene scenario at low CO_2 (EO_450-EO_278).

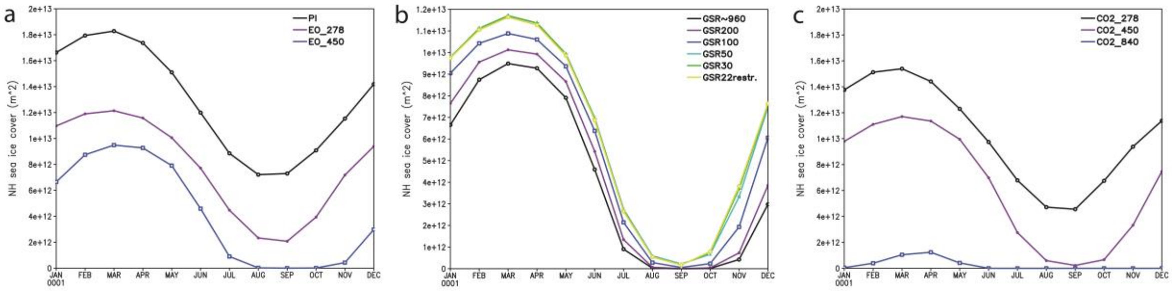

Supplementary Fig. 2: Seasonal cycle of integrated northern hemisphere sea-ice cover for different model scenarios. Panel (a) shows northern hemisohere (NH) sea-ice cover for the Preindustrial model scenario (PI), reduced sea-ice cover (as compared to PI) for the EO scenario at low CO_2 (EO_278) and three months open water conditions for the EO scenario at high CO_2 (EO_450) in the atmosphere. Panel (b) shows the standard EO scenario at 450 ppm CO_2 levels (EO_450, GSR~960) and the effect of limiting the GSR gateway to depth levels of 200 mbsl (GSR200), 100 mbsl (GSR100), 50 mbsl (GSR50), 30 mbsl (GSR30), 22 mbsl and ~370 km gateway width (GSR22restr.). A limited northern ocean heat transport with the return to perennial sea-ice cover is identified at GSR depth levels ~80-100 mbsl. Panel (c) displays the combined effect of GSR gateway depths at 50 mbsl at low (CO2_278), medium (CO2_450) and high (CO2_840) CO_2 levels.

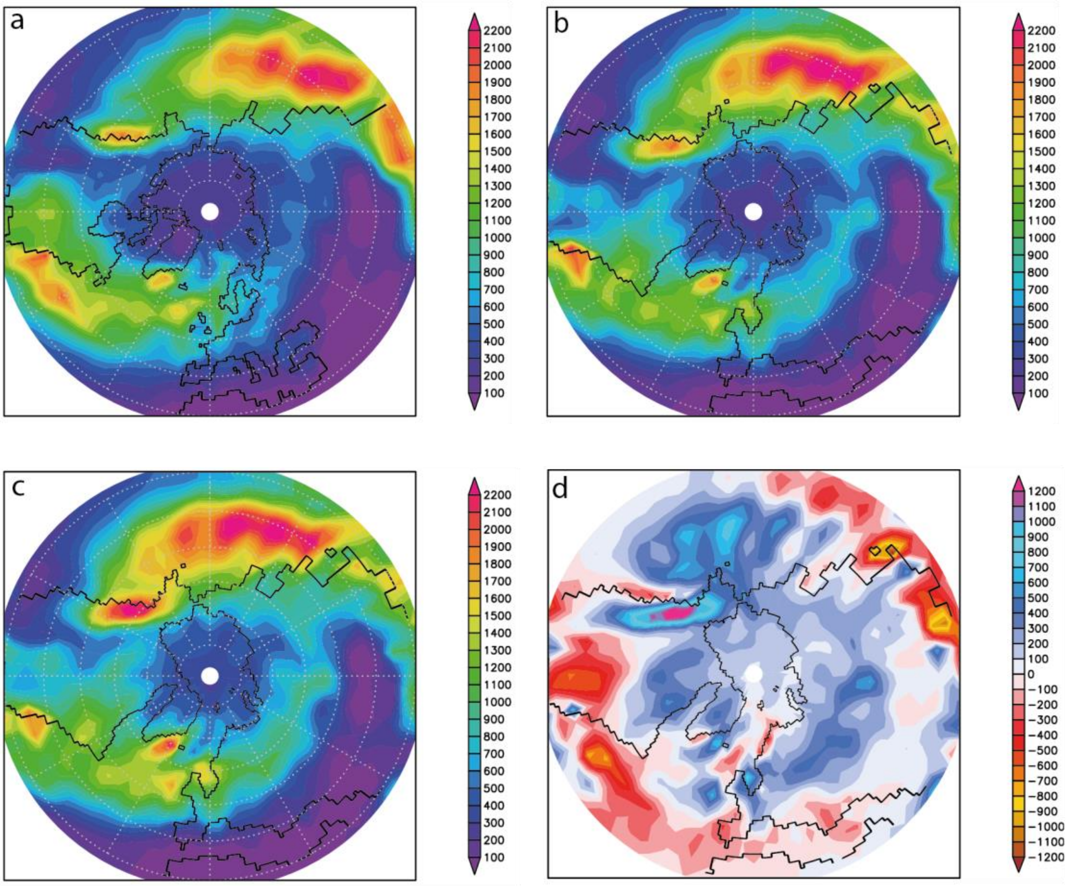

Supplementary Fig. 3: Precipitation for the preindustrial and EO model scenarios. Precipitation (mm·yr^{-1}) for (a) preindustrial ; (b) EO scenario at low CO_2 levels (278 ppm, EO_278); (c) Miocene scenario at high CO_2 levels (450 ppm, EO_450); (d) Anomalous precipitation fields of EO at high CO_2 with respect to the preindustrial scenario (EO_450-PI).

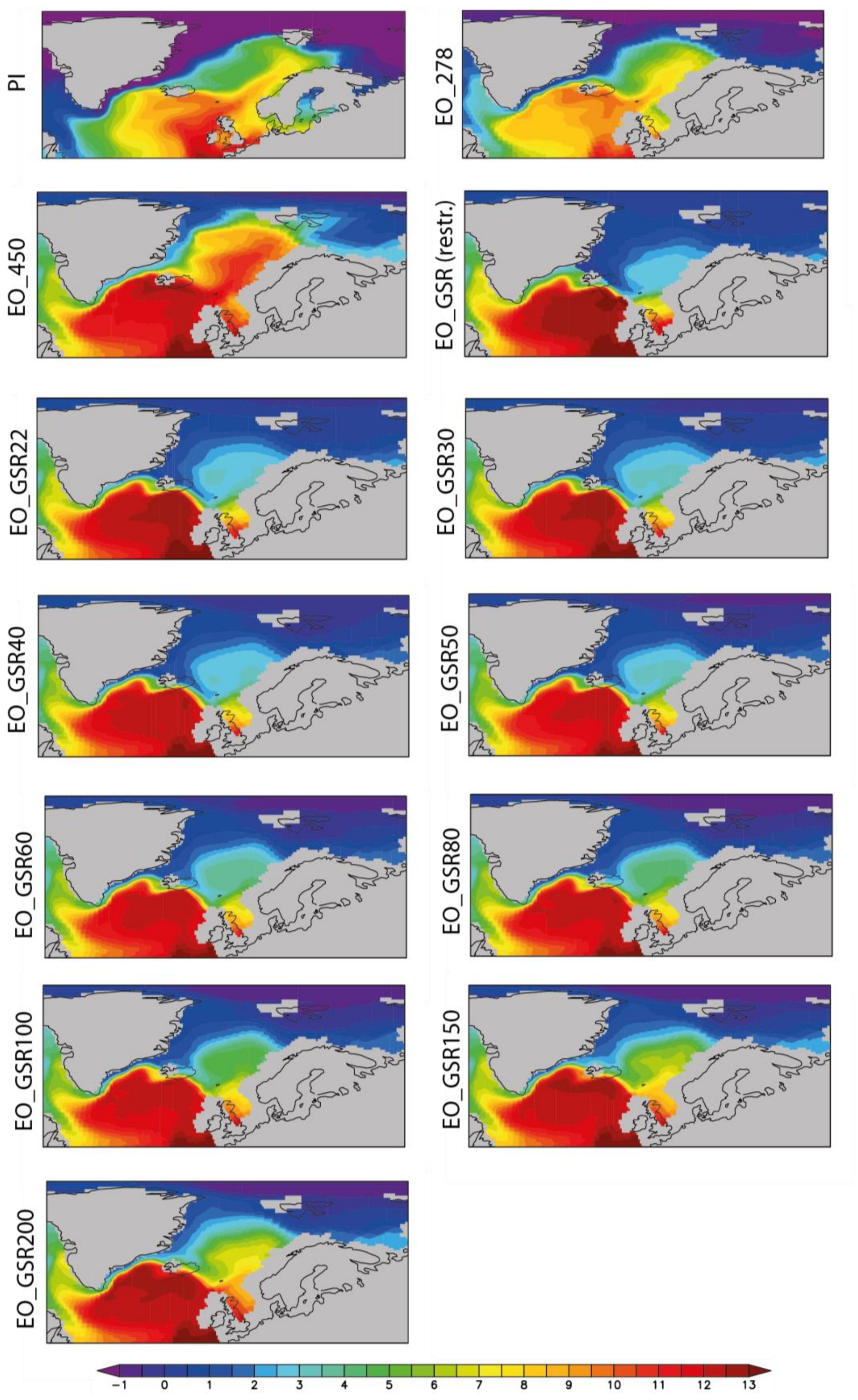

Supplementary Fig. 4: Sea surface temperatures for the Greenland and Norwegian Seas in response to sill depth changes of the Greenland-Scotland Ridge. Sea surface temperature (SST in °C) for preindustrial (PI) and EO model scenarios at 278 ppm CO_2 and 450 ppm CO_2 levels and SST evolution with respect to deepening of the Greenland-Scotland Ridge (GSR, at 450 ppm CO_2).

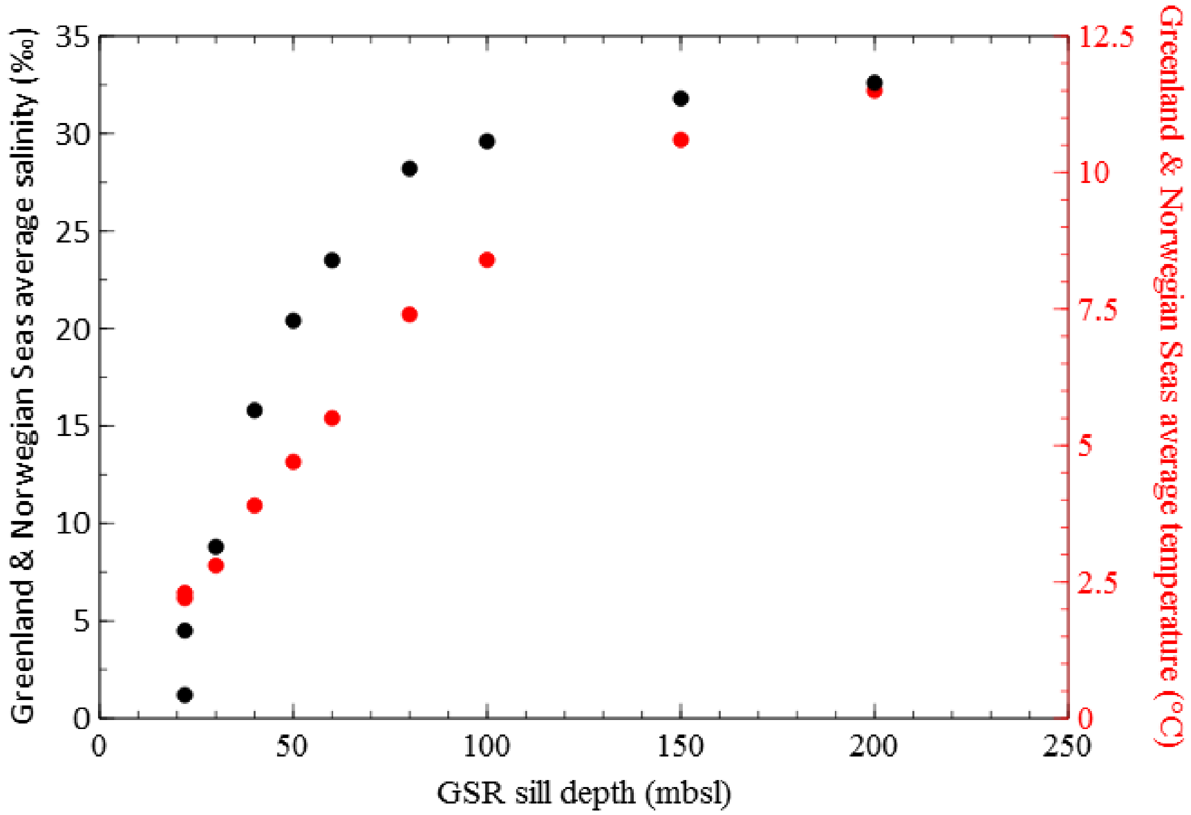

Supplementary Fig. 5: Evolution of integrated mean salinity and temperatures of the Greenland and Norwegian Seas with respect to the GSR sill depth. The mean salinity (‰) is given in black and temperature (°C) in red.

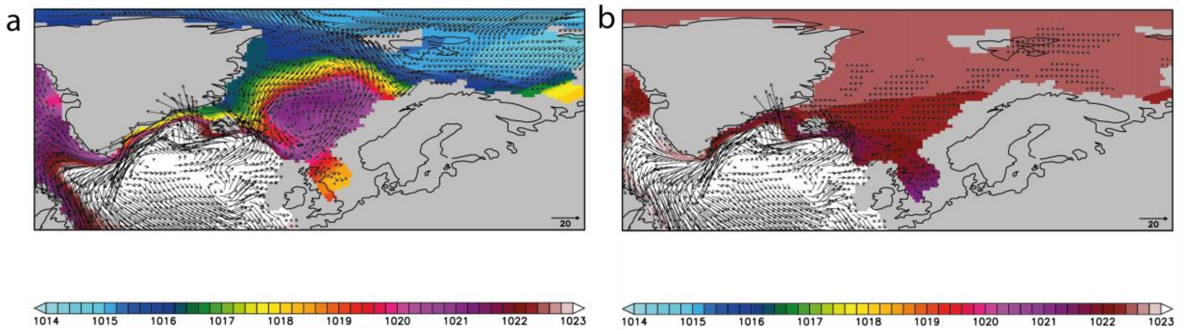

Supplementary Fig. 6: Salinity effect on density calculation and the establishment of ocean currents along density gradients in the surface Arctic Ocean. (a) Density ($kg \cdot m^{-3}$) and ocean currents ($cm \cdot s^{-1}$; velocities $<0.5\ cm \cdot s^{-1}$ are not shown) in the surface Arctic Ocean (6 metres below sea-level) of model scenario EO_GSR100. (b) Same as EO_GSR100, but ocean water density is modelled as a function of temperature and pressure assuming constant salinity (28‰) in the Arctic Ocean.

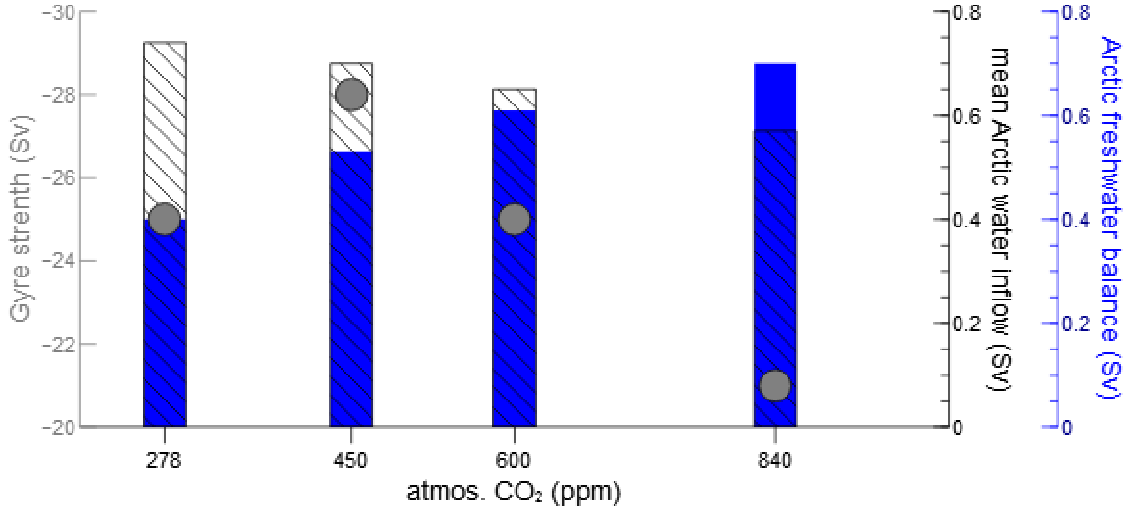

Supplementary Fig. 7: Response of different atmospheric CO_2 levels (in parts per million, ppm) at 50 m sill depth of the GSR on the mean Arctic freshwater balance (incl. net precipitation and river runoff; Sv; blue bars), mean water mass import into the Arctic (Sv; black bars) and the gyre strength (Sv; grey dots) of the Greenland and Norwegian Seas.

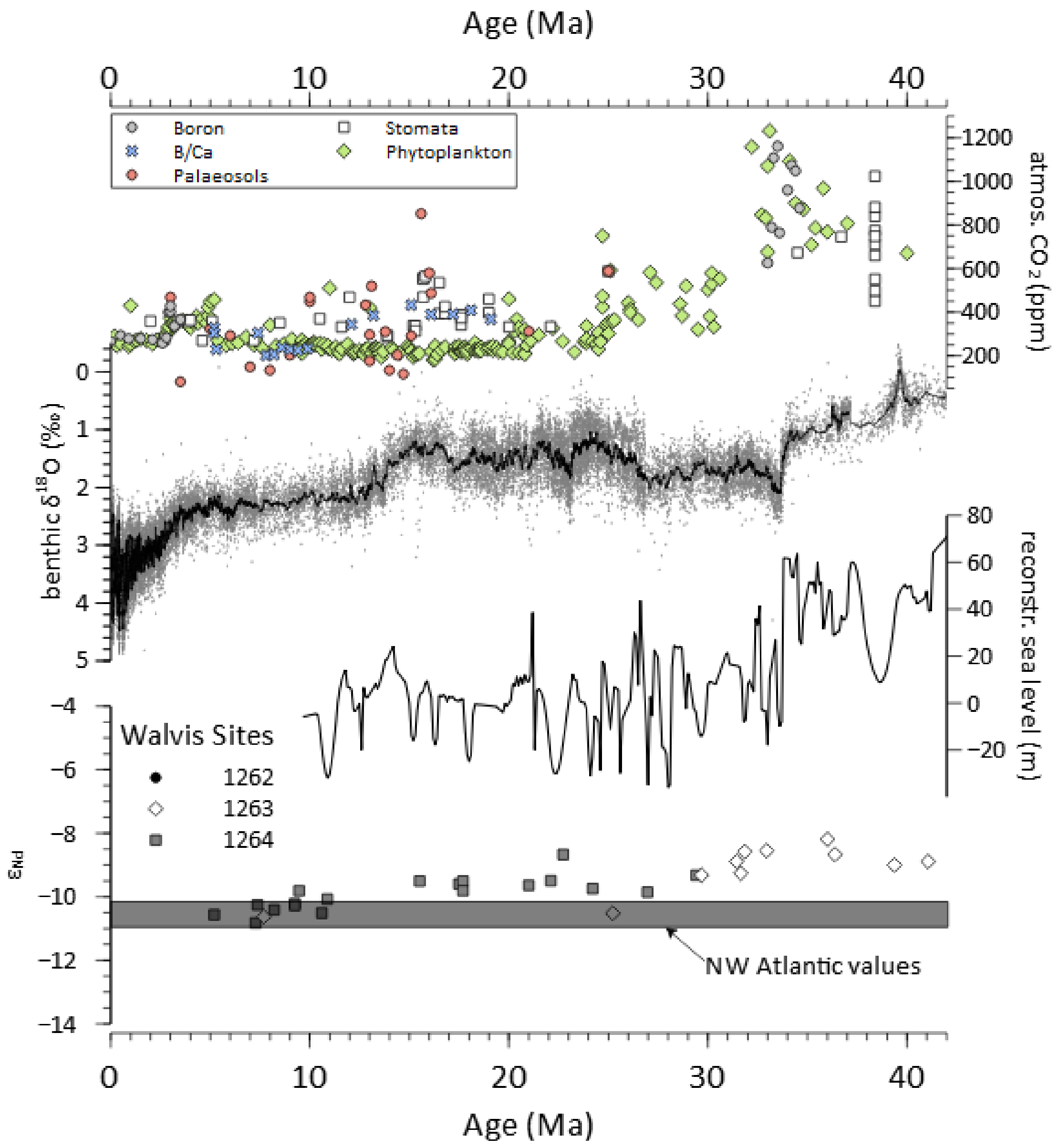

Supplementary Fig. 8: Cenozoic historyfor the last 42 Myrs as displayed by several proxy records. The compilation of proxy records includes CO_2 multi-proxy estimates[1], global stacked benthic $\delta^{18}O$ records[2], reconstructed relative sea-level changes[3] and the neodymium isotope record (ε_{Nd}) from the Walvis sites in the South Atlantic[4].

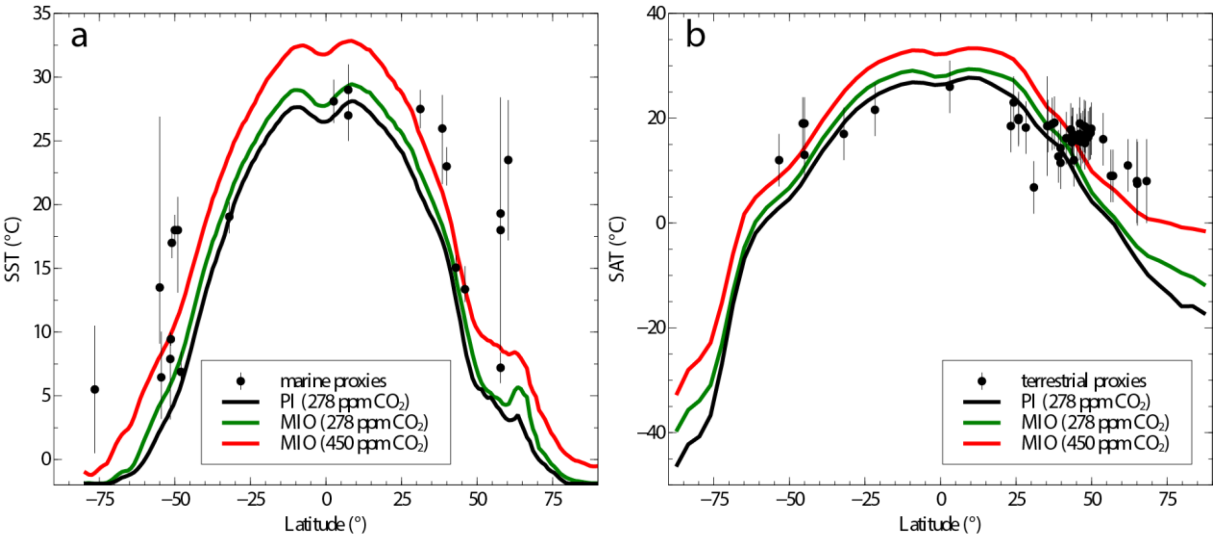

Supplementary Fig. 9: Intercomparison between temperature reconstructions and zonally averaged model data. Proxy data includes errors in methodology as bars (data on proxy and error bars compiled from Krapp and Jungclaus[5]; Goldner et al.[6] and references therein) and the zonally averaged meridional temperature gradient is derived from the Preindustrial (PI) and Miocene model scenarios at low CO_2 (278 ppm, MIO_278) and high CO_2 (450 ppm, MIO_450). (a) Meridional sea surface temperature (SST) compared to marine proxy records; (b) Meridional surface air temperature (SAT) compared to terrestrial proxy records.

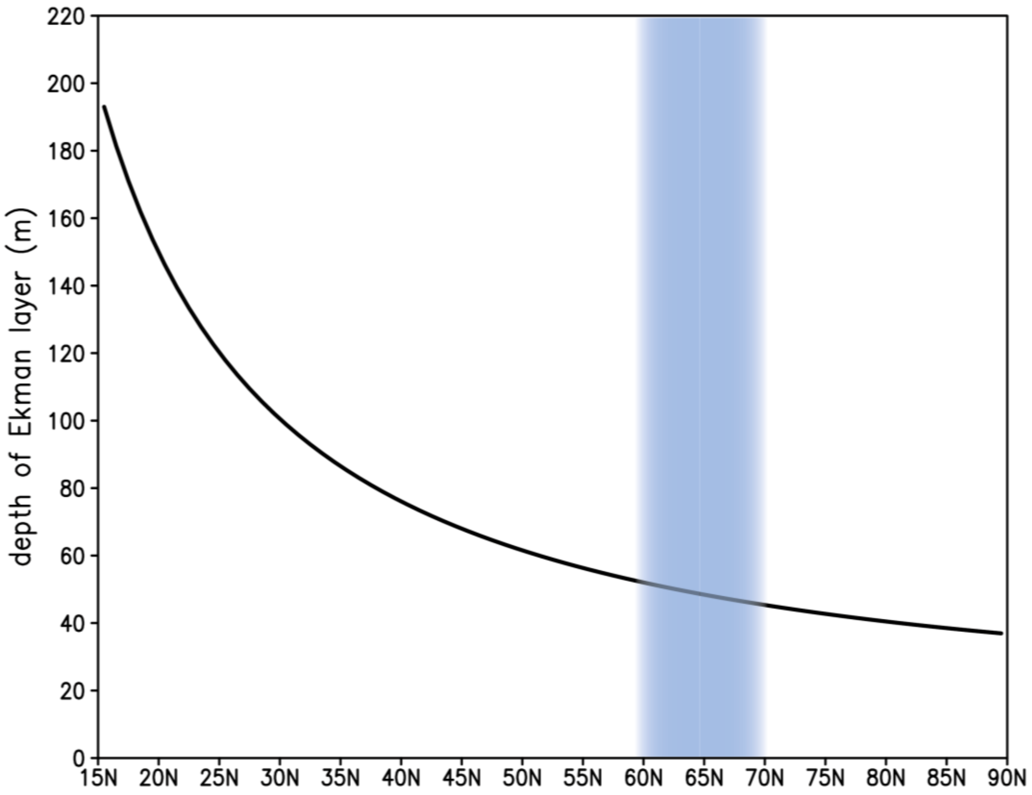

Supplementary Fig. 10: Approximation of the wind-mixed upper ocean layer. The calculation of the depth of frictional influence (depth of Ekman layer in m) is displayed as a function of latitude. The blue bar indicates the approximate latitudinal extent of the Greenland-Scotland Ridge.

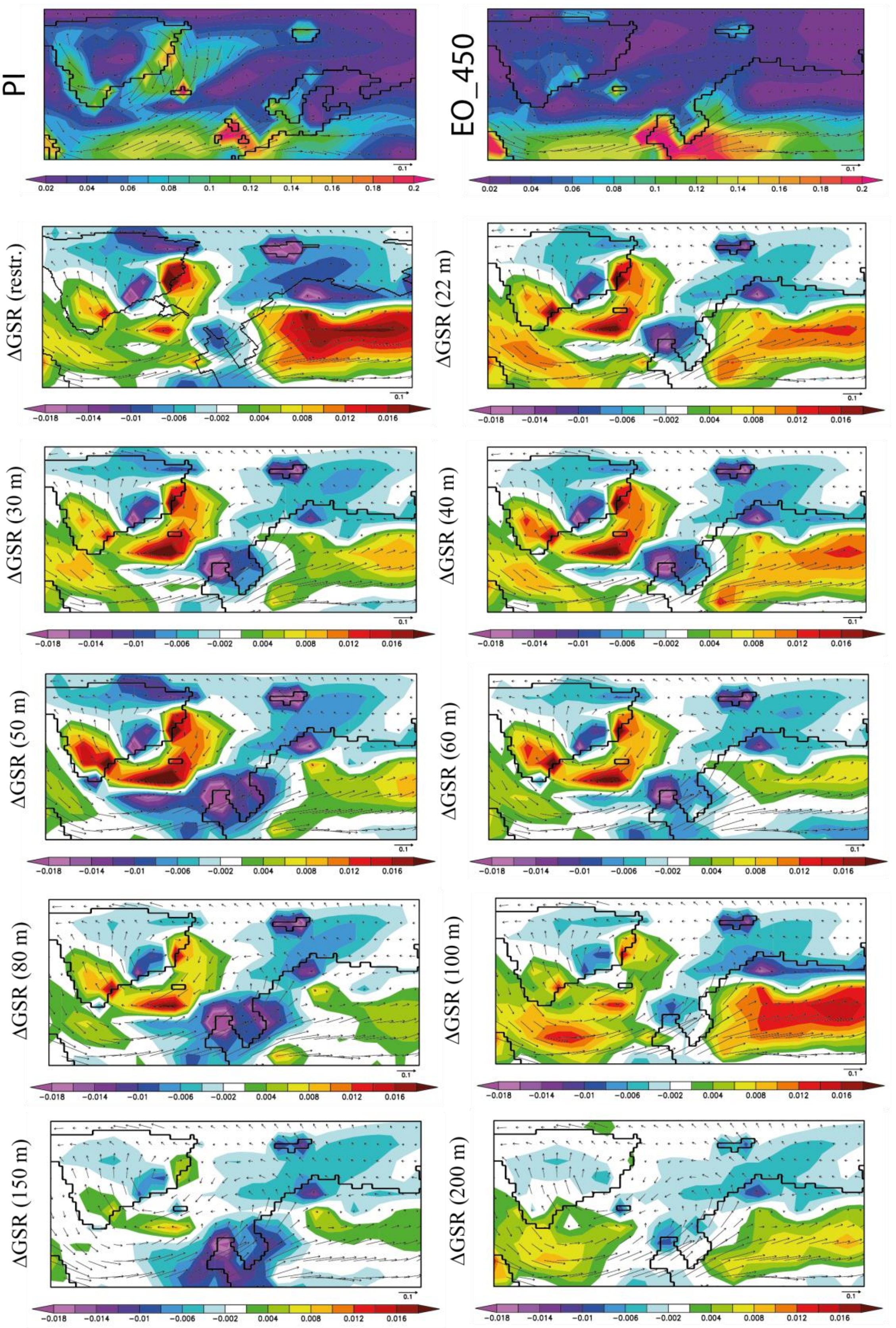

Supplementary Fig. 11: Surface wind stress fields for preindustrial and different Miocene model runs. Surface wind stress fields (N·m^{-2}) for preindustrial (PI), the standard EO scenario (EO_450) and EOT model scenarios at different GSR depth levels. Colour shaded areas indicate the absolute strength of the wind stress (EO_ctl) and anomalies of the GSR sill depth anomalies are compared to EO_ctl (GSR sill depth ~960 m).

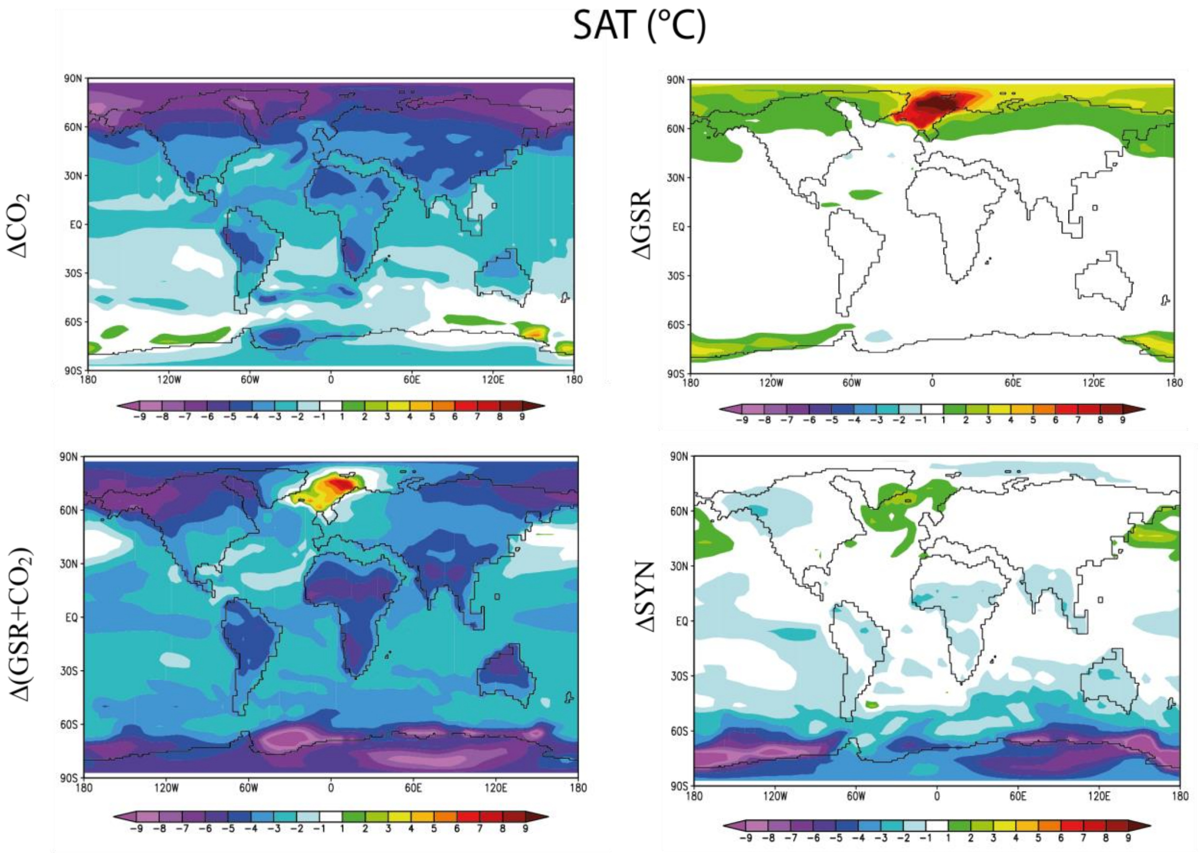

Supplementary Fig. 12: Application of a synergy analysis between atmospheric CO_2 and gateway changes of the Greenland-Scotland Ridge. Synergy analysis (ΔSYN) between declining CO_2 (ΔCO_2), deepening of the GSR (ΔGSR) and the combined effect $\Delta(GSR+CO_2)$ with respect to a change in the surface air temperature (SAT in °C).

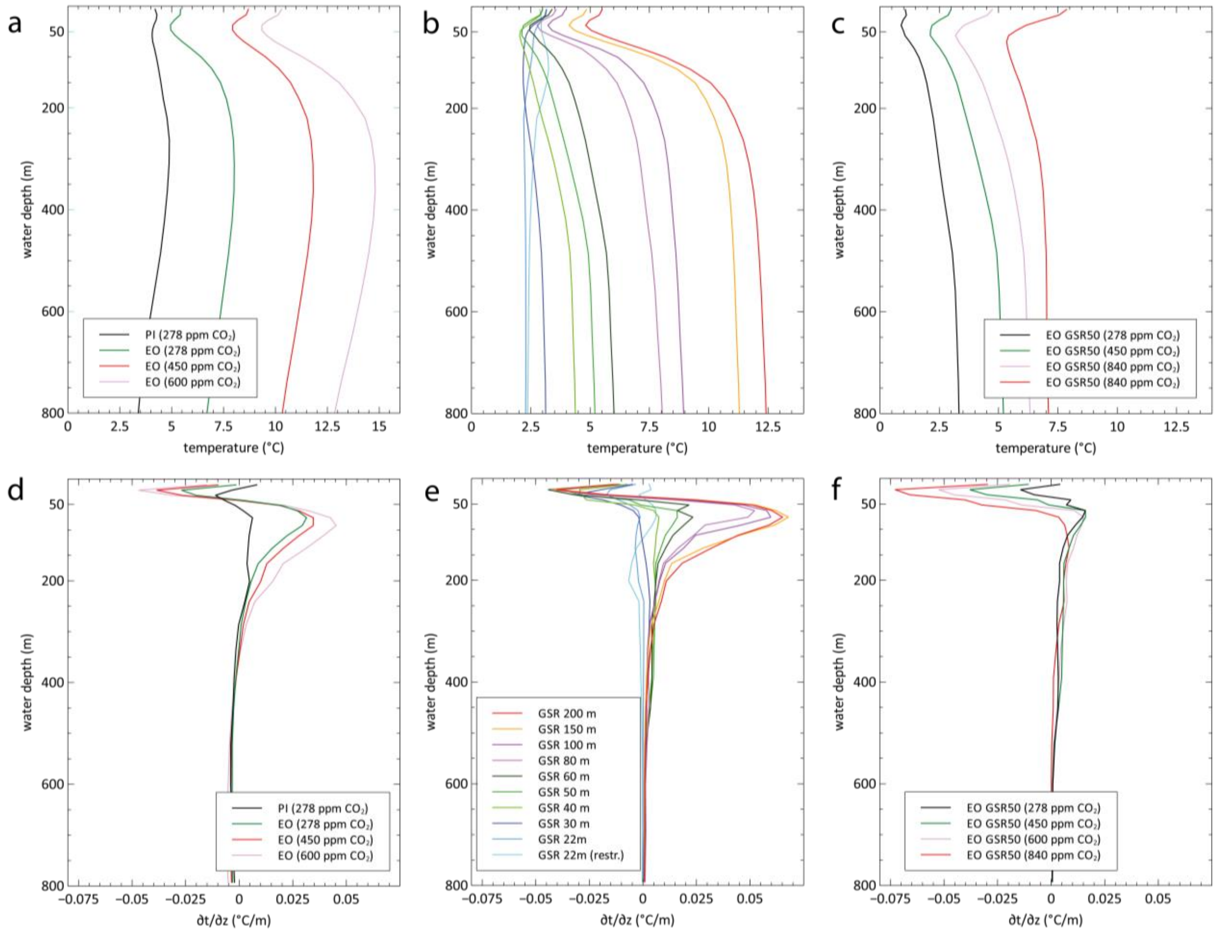

Supplementary Figure 13: Evolution of temperature profiles and the thermocline in the Greenland and Norwegian Seas as a consequence of atmospheric CO_2 changes, gateway changes and the combination of both. Mean ocean temperature profiles (°C) and thermoclines (δt/δz; °C/m) of the subpolar Arctic (Greenland and Norwegian Seas) for different atmos. CO_2 levels (a, d), gateway sill depths (b, e) and different atmospheric CO_2 levels at limited GSR sill depths of 50 metres below sea level (c, f).

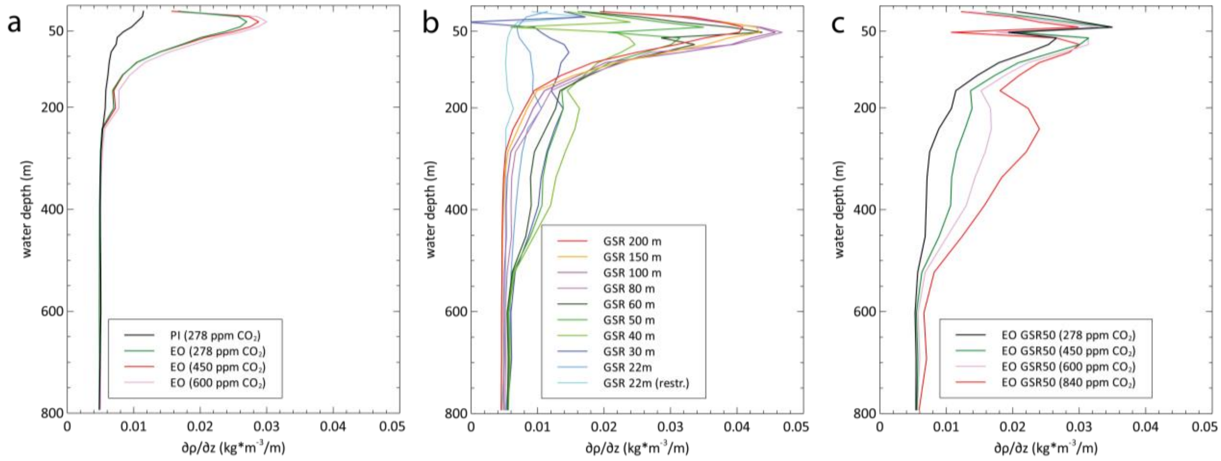

Supplementary Figure 14: Evolution of the pycnocline in the Greenland and Norwegian Seas as a consequence of atmospheric CO_2 changes, gateway changes and the combination of both. Pycnoclines ($\delta p/\delta z$; $kg \cdot m^{-3}/m$) of the subpolar Arctic (Greenland and Norwegian Seas) for different atmospheric CO_2 levels (a), gateway sill depths (b) and different atmos. CO_2 levels at limited GSR sill depths of 50 metres below sea level (c).

Supplementary Table 1: Experimental design and list of the performed model scenarios

Model scenarios	GSR gateway depth (metres below sea-level)	Atmospheric CO_2 (parts per million)	Ocean salinity and temperature initialization	Model integration time (years)
PI	Modern (ca. 1,100)	278	Levitus and Boyer[7]; Levitus et al.[8]	5,500
EO_278	ca. 960	278	Zonal mean[7,8]	4,000
EO_450	ca. 960	450	Zonal mean[7,8]	4,400
EO_600	ca. 960	600	Final 100 yrs mean from EO_450	2,000
EO_GSR22 (restr.)	22 (~370 km gateway width)	450	Final 100 yrs mean from EO_450, Arctic Ocean at 1‰ salinity and 0°C	2,000
EO_GSR22	22	450	Final 100 yrs mean from EO_450, Arctic Ocean at 1‰ salinity and 0°C	2,000
EO_GSR30	30	450	Final 100 yrs mean from EO_450, Arctic Ocean at 1‰ salinity and 0°C	2,000
EO_GSR40	40	450	Final 100 yrs mean from EO_450, Arctic Ocean at 1‰ salinity and 0°C	2,000
EO_GSR50_278 (278 ppm CO_2)	50	278	Final 100 yrs mean from EO_278, Arctic Ocean at 1‰ salinity and 0°C	2,000
EO_GSR50 (450 ppm CO_2)	50	450	Final 100 yrs mean from EO_450, Arctic Ocean at 1‰ salinity and 0°C	2,000
EO_GSR50_600 (600 ppm CO_2)	50	600	Final 100 yrs mean from EO_450, Arctic Ocean at 1‰ salinity and 0°C	2,000
EO_GSR50_840 (840 ppm CO_2)	50	840	Final 100 yrs mean from EO_450, Arctic Ocean at 1‰ salinity and 0°C	2,000
EO_GSR60	60	450	Final 100 yrs mean from EO_450, Arctic Ocean at 1‰ salinity and 0°C	2,000
EO_GSR80	80	450	Final 100 yrs mean from EO_450, Arctic Ocean at 1‰ salinity and 0°C	2,000
EO_GSR100	100	450	Final 100 yrs mean from EO_450, Arctic Ocean at 1‰ salinity and 0°C	2,000
EO_GSR150	150	450	Final 100 yrs mean from EO_450, Arctic Ocean at 1‰ salinity and 0°C	2,000
EO_GSR200	200	450	Final 100 yrs mean from EO_450, Arctic Ocean at 1‰ salinity and 0°C	2,000

Supplementary Table 2: Climate variables for specific model scenarios

model scenarios	Integr. net freshwater flux (net precip. + river runoff) into Arctic Ocean (Sv)	NADW index (Sv)	AABW index (Sv)	Northern hemisphere sea-ice volume ($10^3 \cdot km^3$)	mean SSS Arctic Ocean (‰)	mean SST Arctic Ocean (°C)	global mean SAT (°C)	global mean precip ($mm \cdot yr^{-1}$)	global mean vert. Integr water vapor ($kg \cdot m^{-2}$)	mean salinity of Arctic Ocean(‰)	mean ocean temperature of Arctic Ocean (°C)	calculated from $\delta^{18}O$-salinity relationship[9]		
												mean $\delta^{18}O$ values in the Arctic Ocean	Expected global $\delta^{18}O$ changes (‰) in the seawater based on volume constraints of ocean basins:	
													Present day	Miocene bathymetry reconstr.[10]
**PI**	0.25	16.8	-3.3	31.6	31.3	-0.28	14.4	996	25.2	34.7	2.2	-0.16	0.00	XXX
**EO_278**	0.56	11.7	-1.7	9.9	25.4	0.6	16.8	1038	29.5	34.4	5.1	-0.31	0.00	0.00
**EO_450**	0.67	11.8	-0.5	3.8	24.5	0.24	20.4	1115	38	34.3	7.7	-0.36	0.00	-0.01
**EO_600**	0.60	10.8	-0.4	1.2	24	3.9	22.5	1155	44.1	34.3	9.4	-0.36	0.00	-0.01
**EO_GSR22 restr. (~370km width)**	0.70	11.2	-1.8	6.8	1	1	20.7	1120	38.8	1.1	2.0	-16.3	-0.23	-0.26
**EO_GSR22**	0.69	11.3	-0.9	7.0	2	1.1	20.4	1114	38.2	4.4	2.0	-14.7	-0.21	-0.23
**EO_GSR30**	0.68	10.7	-0.8	6.9	4	1.1	20.3	1112	37.9	8.9	2.8	-12.6	-0.16	-0.20
**EO_GSR40**	0.68	10.6	-0.7	7.2	8	0.9	20.1	1107	37.4	15.9	3.9	-9.2	-0.13	-0.15
**EO_GSR50_278**	0.55	8.2	-6.5	19	13.7	-0.18	17.5	1052	30.6	21.1	3.1	-6.7	-0.09	-0.11
**EO_GSR50_450**	0.67	10.2	-1.1	7.0	11.6	0.9	20.1	1107	37.4	20.4	4.7	-7.0	-0.10	-0.11
**EO_GSR50_600**	0.61	9.9	-0.47	5.4	10	2.0	22.3	1151	43.8	19.8	5.7	-7.3	-0.10	-0.12
**EO_GSR50_840**	0.87	6.6	-0.9	0.1	7.9	5.1	25.0	1214	52.5	18.5	6.5	-8.0	-0.11	-0.13
**EO_GSR60**	0.68	9.9	-0.9	6.5	14.1	0.9	20.3	1111	37.8	23.4	5.4	-5.6	-0.08	-0.09
**EO_GSR80**	0.67	10.9	-0.6	6.1	18	0.9	20.2	1109	37.6	28.1	7.4	-3.3	-0.05	-0.05
**EO_GSR100**	0.68	11.1	-0.6	5.8	19.4	1	20.2	1110	37.7	29.5	8.3	-2.7	-0.04	-0.04
**EO_GSR150**	0.69	11.4	-1.4	4.8	21.2	1.3	20.7	1120	38.7	31.7	10.6	-1.6	-0.04	-0.03
**EO_GSR200**	0.69	11.6	-1.6	4.6	21.7	1.5	20.7	1120	38.7	32.4	11.4	-1.3	-0.02	-0.02

Supplementary References

1. Beerling, D. J. & Royer, D. L. Convergent Cenozoic CO_2 history. Nat. Geosci. 4, 418–420 (2011).
2. Cramer, B. S., Toggweiler, J. R. , Wright, J. D., Katz, M. E. & Miller, K. G. Ocean overturning since the Late Cretaceous: Inferences from a new benthic foraminiferal isotope compilation. Paleoceanography 24, PA4216, doi:10.1029/2008PA001683 (2009).
3. Kominz, M. A. , Browning, J. V., Miller, K. G., Sugarman, P. J., Mizintseva, S. & Scotese, C. R. Late Cretaceous to Miocene sea-level estimates from the New Jersey and Delaware coastal plain coreholes: an error analysis. Basin Res. 20, 211–226 (2008).
4. Via, R. K. & Thomas, D. J. Evolution of Atlantic thermohaline circulation: Early Oligocene onset of deep-water production in the North Atlantic. Geology, 34, 441–444 (2006).
5. Krapp, M. & Jungclaus, J. H. The Middle Miocene climate as modelled in an atmosphere-ocean-biosphere model. Clim. Past, 7, 1169-1188, doi:10.5194/cp-7-1169-2011 (2011).
6. Goldner, A., Herold, N. & Huber, M. The challenge of simulating the warmth of the mid-Miocene climatic optimum in CESM1. Clim. Past 10, 523–536, doi:10.5194/cp-10-523-2014 (2014).
7. Levitus, S. & Boyer, T. P. World Ocean Atlas 1994, Vol. 4. Temperature, NOAA Atlas NESDIS 4, U.S. Gov. Printing Office, Washington, D.C., 117 pp (1994).
8. Levitus, S., Burgett, R. & Boyer, T. World Ocean Atlas 1994, Vol. 3: Salinity. NOAA Atlas NESDIS 3, U.S. Gov. Printing Office, Washington, D.C., 99 pp (1994).

9. LeGrande, A. N. & Schmidt, G. A. Global gridded data set of the oxygen isotopic composition in seawater. Geophys. Res. Lett. 33, L12604, doi:10.1029/2006GL026011 (2006).

10. Herold, N., Seton, M., Müller, R. D., You, Y. & Huber, M. Middle Miocene tectonic boundary conditions for use in climate models. Geochem Geophys Geosyst 9, Q10009, doi:10.1029/2008GC002046 (2008).
